# Hi-C-resolved metagenomics reveals host range variation among mobile genetic elements within the European honey bee

**DOI:** 10.1128/mbio.02243-25

**Published:** 2025-09-22

**Authors:** Chris R. P. Robinson, Adam G. Dolezal, Ivan Liachko, Irene L. G. Newton

**Affiliations:** 1 Department of Biology, Indiana University 1772, Bloomington, Indiana, USA; 2 University of Illinois Urbana-Champaign 14589, Urbana, Illinois, USA; 3 Phase Genomics 581436 , Seattle, Washington, USA; University of Hawaii at Manoa, Honolulu, Hawaii, USA

**Keywords:** plasmids, antibiotic resistance, metagenomics, honey bee, symbiosis

## Abstract

Mobile genetic elements (MGEs), such as plasmids and bacteriophages, are major contributors to the ecology and evolution of host-associated microbes due to symbiotic interactions and gene flow via horizontal gene transmission. Antibiotic resistance genes (ARGs), which are frequently trafficked via MGEs, are known to be enriched within North American honey bee microbiomes due to decades of antibiotic exposure. While previous studies have identified nearly identical MGE-associated ARGs across geographically disparate honey bee colonies, our understanding of how ARGs are distributed and mobilized within and between individual microbiomes is limited. To address this limitation, we leverage Hi-C-resolved metagenomics with the honey bee worker gut microbiome and show that the worker gut contains dense, nested, and highly distinct MGE communities. We show that phage-microbe networks exhibit high variation among individual metagenomes and that phages show broad host range with respect to both the number and phylogenetic distance of their hosts. Comparisons of individual microbiomes reveal highly individualized plasmid communities that exhibit broad host range variation within microbiomes. Finally, we provide specific evidence that antibiotic resistance cassettes are being actively shuttled between honey bee microbes via plasmids and that these broad host range plasmids frequently recombine to share gene content. Our work corroborates early observations of ARG dispersal in honey bee colonies and provides evidence for how these genes are mobilized within and across honey bee-associated microbial communities.

**IMPORTANCE:**

Mobile genetic elements (MGEs) are found in every microbial community and often encode genes conferring antibiotic resistance (ARGs). Within the honey bee worker gut microbiome, ARGs are particularly frequent due to decades of antibiotic exposure. Previous studies have identified nearly identical ARGs in geographically disparate honey bee colonies, which suggests recent mobilization by MGEs into these colonies, but identifying how these ARGs are mobilized and distributed within honey bee colonies remains a challenging task, as most techniques rely on microbial culture. Applying metagenomic Hi-C, we describe how these ARGs are distributed among individual plasmid backbones and how those plasmids are distributed among host microbial populations. Remarkably, we find plasmids exhibit broad host range variation, although they encode nearly identical ARGs. Our work corroborates earlier observations of ARG dispersal in honey bee colonies and provides further evidence for how these ARGs are mobilized across vast geographic distance.

## INTRODUCTION

Mobile genetic elements, or MGEs, are at the center of ongoing environmental and health-related crises due to their ability to transfer genes—including those conferring antibiotic resistance—via mechanisms of horizontal gene transmission (HGT). Plasmids and bacteriophages are among the most important classes of MGEs due to their ability to mediate conspecific and interspecific HGT. These two classes are often embedded within dense microbial communities and exhibit high variation in transmission rate (1) and gene content (2), yet most interactions between microbial hosts and MGEs are studied in homogeneous liquid cultures (3), which limit our understanding of the factors that influence MGE-encoded trait variability. The ecological and evolutionary impact of MGEs within natural microbial communities is far less understood. For example, phages can drive changes in microbial community composition (4–8), but evidence for this is inconsistent (9–12). Alternatively, the HGT-mediated transmission of genes often results in low genetic linkage among MGE-encoded genes, resulting in high gene flux. Variation in HGT rate among MGEs—and the genes they encode—likely plays a critical role in MGE evolution.

Recent work in several natural systems (5, 13–16) has shown that viruses likely associate with several phylogenetically distant microbial hosts. Similarly, groups of highly similar plasmids have been shown to exhibit tremendous variation in host range (17). The stability and frequency of these interactions are thought to be mediated by host population diversity, community diversity, and transmission rate (18–22). In turn, variation at the level of MGE transmission rate has been shown to affect the evolution of phage virulence (23) and impact the gene content carried by plasmid replicons (18, 24). However, MGE-associated host range variation has only been examined in a handful of systems, and our understanding of whether and how this variation might impact functional traits relevant to associated microbial hosts remains to be determined. A powerful model system with which to explore MGE dynamics within host-associated microbial communities is the Western honey bee (*Apis mellifera*), due to its long co-evolutionary history with its gut microbiome (25), which has resulted in a stable, relatively low complexity microbial community. A small number of studies have explored bee-associated phage communities (26–31) and plasmid communities (32, 33). Studies that have investigated bee-associated phages have found that honey bees are associated with a highly diverse phage community that exhibits high variability in phage population sizes and rates of co-evolution with microbial hosts. CRISPR-based methods suggest that *Gilliamella*, *Lactobacillus*, and *Bombilactobacillus* are among the most frequently predicted hosts for bee-associated phages (26–28). However, compared to studies of bee phages, there have been far fewer metagenomic analyses of the bee-associated plasmid community. Between 1950 and 2005, oxytetracycline was routinely applied to honey bee colonies to control the bacterial disease larval foulbrood, and recent studies have found that this treatment resulted in a high frequency of antibiotic resistance genes (ARGs) in the honey bee microbiome (32, 33). Surprisingly, both studies recovered nearly identical ARGs from geographically disparate bee colonies and show that plasmids likely facilitate the transfer of these ARGs between colonies. However, while both studies were able to indirectly link ARGs to putative plasmid association via co-occurrence with plasmid hallmark genes, these studies were unable to directly link these genes to individual plasmid backbones due to methodological limitations, nor could they connect these contigs to microbial hosts. Understanding how individual ARGs are partitioned among distinct plasmids, and how those plasmids are distributed among bacterial hosts within the honey bee gut, remains open questions.

Here, we sought to comprehensively understand viral and plasmid dynamics within the honey bee worker gut microbiome. We hypothesized that both viruses and plasmids with variable host ranges (e.g., multiple species) will be prevalent within honey bee worker microbiomes. Because of the long history of antibiotic treatment of honey bees in agricultural settings, we further hypothesized that these MGEs might mediate the transmission of antibiotic resistance between microbial hosts. We also hypothesized that ecological interactions between microbes might facilitate the transmission of MGEs between distantly related genera. To test these hypotheses, we conducted deep metagenomic and Hi-C proximity ligation sequencing of three age-matched honey bee worker pools, each originating from one of three colonies governed by a single drone-inseminated queen and held in the same apiary. We show that colonies harbor similar microbial communities and plasmids (via relaxase and replicon typing), while phage communities are highly heterogeneous and specific to each colony. We identify near-identical and syntenic plasmid-associated gene modules across all three honey bee colonies, suggesting either plasmid transmission between colonies or the recent acquisition of genes by individual plasmids. By examining Hi-C networks, we show that phage host range is dynamic within honey bee worker microbiomes and that phage conspecifics exhibit wide variation in the number and phylogenetic diversity of their microbial hosts. We show that plasmids, not phages, are mediating the horizontal acquisition of antibiotic resistance in the honey bee microbiome. Finally, we show that phages likely interact with distantly related microbes within the honey bee gut and frequently target hosts known to form a biofilm in the ileum. Using the honey bee model to study natural microbial–MGE dynamics *in vivo*, our results show that the gut microbiome contains a highly diverse MGE community that exhibits a wide range of host range variation among microbial hosts and highlights how this variation may affect both microbial and MGE evolution in a natural animal-associated microbial community. Our work corroborates early observations of near-identical ARGs from geographically distant honey bee colonies and advances our understanding of how these ARGs are distributed both within and across honey bee colonies.

## RESULTS

### Recovery of core bacterial phylotypes from the honey bee gut

**Fig 1:**
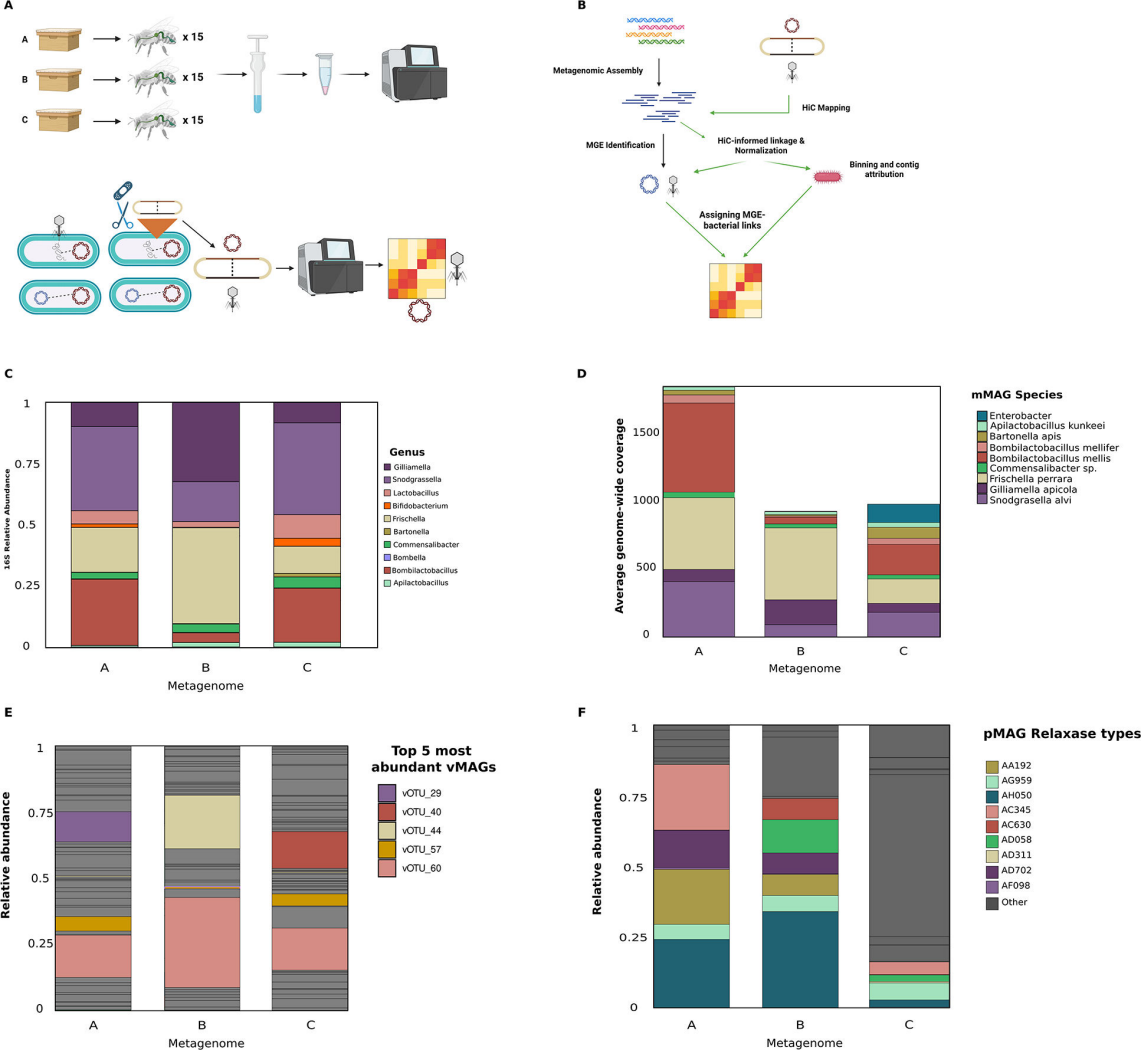
Core honey bee microbial taxa recovered from metagenomic sequencing. (**A**) Graphical overview of honey bee sampling, dissection, and sequencing (top) and metagenomic Hi-C sequencing (bottom). (**B**) Graphical overview of major steps of bioinformatic workflow for metagenomic assembly and MGE host association via mapping of chimeric reads generated during Hi-C workflows. (**C**) 16S rRNA gene-based relative abundance of core and common microbial genera within the honey bee worker microbiome. Individual taxa are color-coded by genus. (**D**) Average genome-wide coverage of dereplicated and representative high-quality microbial genomes recovered during assembly. Genomes are color-coded by taxonomy. (**E**) Relative abundance of phage vMAGs within each metagenome. The top five most abundant vMAGs are colored. (**F**) Relative abundance of top five most abundant plasmids across all metagenomes. Plasmids are color-coded by relaxase identification. Shared colors indicate plasmids recovered with similar relaxase genes but do not represent identical plasmids.

We performed metagenomic and Hi-C sequencing of three pools of honey bee workers (Fig. 1A and B for graphical overview of sequencing and analytical methods). Each pool consisted of dissected guts from 15 age-matched workers, collected from one of three healthy, single-drone inseminated colonies, housed at the same apiary (the Bee Research Facility at University of Illinois at Urbana-Champaign). Microbes were enriched through differential centrifugation from these pools and were used for gDNA extraction (see Methods). gDNA was used for metagenomic sequencing, yielding a total of 166,031,286; 131,589,634; and 126,650,820 150 bp read pairs across metagenomes A, B, and C, respectively. The majority of studies of the honey bee microbiome quantify the abundance of core taxa via 16S rRNA gene quantification. To ensure that our metagenomic data were in line with these studies, we quantified the relative abundance of known bee microbial phylotypes by mapping metagenomic reads to BEExact, which represents a curated database for 16S rRNA gene-based analyses (34) (Fig. 1C). As found in other 16S rRNA gene-based surveys of the honey bee worker gut, we recovered similar core and common microbial bee-associated genera across each individual metagenome, although the relative abundance of each genus varied between each sample. From this initial screen, we recovered the genera *Gilliamella*, *Snodgrassella*, *Lactobacillus*, *Bifidobacterium*, *Frischella*, *Bartonella*, *Commensalibacter*, *Bombella*, *Bombilactobacillus*, and *Apilactobacillus*. Since our 16S rRNA gene-based analyses recovered all core taxa within the honey bee worker gut, we next assembled and binned microbial MAGs from each of the metagenomes

by combining data from multiple binning strategies and from information gleaned from Hi-C-inferred contig-to-contig linkages (see Methods). Overall, we recovered 28 genomes from our binning strategy, including 4 *Apilactobacillus*, 3 *Bartonella*, 5 *Bombilactobacillus*, 1 *Enterobacter*, 2 *Bifidobacterium*, 1 *Frischella*, 3 *Lactobacillus*, 3 *Commensalibacter*, 3 *Snodgrassella*, and 3 *Gilliamella*. However, only 13 of these genomes met the completeness (*≥*50%) and contamination (*≤*10%) thresholds based on the Genomic Standards Consortium (35). A major methodological goal in our study was to reduce the degree of spurious Hi-C interactions between MGEs and their host bacteria since highly contaminated assemblies were more likely to report incorrect interactions due to a higher level of extraneous contigs. Although the removal of low-quality genome assemblies—especially of core microbial taxa—may result in a loss of information in our data set, the inclusion of contaminated assemblies would produce untrustworthy results. We therefore chose to proceed with the 13 best MAGs. The 13 microbial genomes that passed our quality thresholds were dereplicated at 97% ANI and 85% breadth, then used to recruit reads from all three metagenomes.

Nine microbial metagenome-assembled genomes (mMAGs) were retained following dereplication and represent four core phylotypes (*Gilliamella apicola*, *Snodgrassella alvi*, *Bombilactobacillus mellifer*, and *Bombilactobacillus mellis*) and five non-core phylotypes (*Commensalibacter*, *Frischella*, *Enterobacter*, *Apilactobacillus*, and *Bartonella*) of the honey bee worker microbiome. The average genome-wide coverage of these high-quality mMAGs was comparable across metagenomes, except for *Enterobacter*, which was more abundant in metagenome C (Fig. 1D). Recovered mMAGs represent the majority of observed phylogenetic diversity within the honey bee worker gut microbiome (36), although we note that the removal of contaminated *Lactobacillus* and *Bifidobacterium* genome assembles limits our ability to observe MGE interactions across all core microbial taxa within the honey bee gut. However, these organisms were likely in lower abundance in our samples, with *Lactobacillus* accounting for 5.39%, 2.27%, and 9.56% of 16S rRNA gene abundance in metagenomes A, B, and C, while *Bifidobacterium* accounted for only 1.43%, 1%, and 3.26% in metagenomes A, B, and C, respectively (Fig. S1). Finally, we mapped reads to a database containing the honey bee genome, mMAGs, pMAGs, and vMAGs (discussed below) and found that only 12.7%, 33.6%, and 12.9% of reads mapped to the honey bee genome in metagenomes A, B, and C, respectively (Fig. S2). The bulk of the remaining reads were attributed to assembled MAGs. The average coverage to each mMAG contig across each metagenome is provided in Table S1.

**Fig 2:**
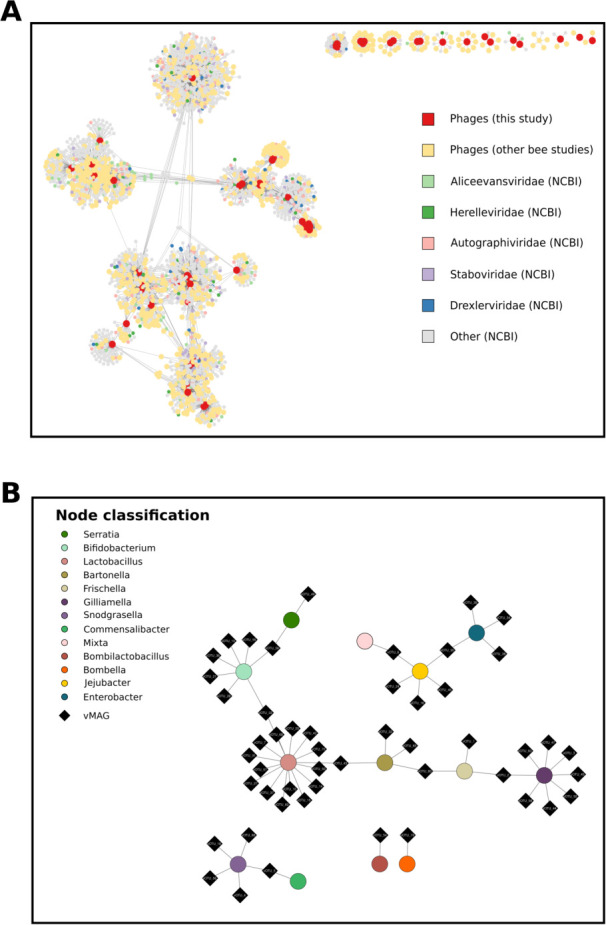
Recovered honey bee phages cluster with existing bee phages and are targeted by core microbial taxa. (**A**) vConTACT3 network generated from phage genomes recovered from this study (red), from all other bee phage studies where viral genomes were available (yellow), and from NCBI RefSeq (gray). The five viral families exhibiting the most connectivity to bee phages are colored *Aliceevansviridae* (light green), *Herelleviridae* (dark green), *Autographiviridae* (pink), *Staboviridae* (lavender), and *Drexelviridae* (blue). (**B**) iPHoP host-phage association network generated via high-confidence predictions. Black diamonds represent high-quality phage genomes, while circles represent predicted hosts as inferred from GTDB. Hosts are color-coded by taxonomy.

### Honey bee colonies host specific phage communities but share plasmids with similar relaxase genes

Having characterized the putative microbial hosts within the microbiome of our samples, we next explored the presence and distribution of plasmids due to their association with ARGs in the honey bee worker microbiome. Our investigation of the honey bee worker gut plasmidome led to the recovery of a total of 41 plasmids from across all metagenomes. Due to the challenges inherent to plasmid assembly from short metagenomic reads and the high similarity between bacterial and plasmid-associated genes due to HGT, we used a previously benchmarked plasmid discovery pipeline (37). First, plasmid sequences were called and clustered via MOB-suite (37), which uses a reference database containing sequences of known plasmid origin. We restricted our analysis to the recovery of these highly complete, high-confidence plasmids (pMAGs). Plasmid sequences within each metagenome were then dereplicated at 95% ANI across 85% of the plasmid length and compared against mMAG contigs to control for incorrect inclusion of pMAG contigs in mMAG assemblies. We note that while our approach is intended to reduce the instances of false positives in plasmid discovery, it likely underestimates the observed plasmid diversity in the honey bee worker gut metagenome. pMAGs varied greatly in size, exhibiting an average length of 9.7 kb (min. 2.2 kb to max. 55 kb). A total of 12/42 recovered pMAGs encoded genes for plasmid mobility, either through predicted mobilization via another MGE (*n* = 11, encoding their own relaxase) or by encoding their own mechanism for conjugation (*n* = 1, encoding a full conjugation machinery cassette). pMAGs were classified via MOB-suite relaxase typing. Several relaxase types were found in all three metagenomes (three relaxase types) and two of the three metagenomes (six relaxase types). The remaining pMAG relaxase types were unique to each metagenome (Fig. 1D).

Next, we characterized the phage community within each metagenome. Overall, we recovered 4,632 viral contigs, although the majority of these contigs represent low-confidence predictions. Removal of low-quality phage genomes is standard practice in studies of viral ecology and evolution. After an additional filtering step, we retained 77 phage genomes classified by CheckV as complete (*n* = 8), high-quality (*n* = 27), or medium-quality (*n* = 42) (38). We note that our decision to use phage, plasmid, and microbial genomes above a certain quality threshold reflects both community standards for metagenomic assembly (35) and our goal to reduce spurious Hi-C contacts between MGEs and microbial hosts.

Recovered phage genomes were dereplicated at 95% ANI and 85% breadth down to 70 genomes (vMAGs), which were used as representative viral genomes for all downstream analyses. Our dereplication strategy reflects known species-equivalent OTU delineations in other bacteriophage communities (39–42) and resulted in only a 9% reduction in the number of viral genomes, suggesting a large degree of vMAG genomic variation across the three metagenomes. Similar to pMAG size, vMAG

genome size was highly variable, averaging 28 kb (min. 3 kb, max. 143 kb) and encoding an average of 35 genes. A small subset (*n* = 5) of vMAGs was called putatively proviral by CheckV. The relative abundance of vMAGs between metagenomes was highly heterogeneous (Fig. 1E), exhibiting extensive variability relative to the recovered pMAG relaxase types.

To further characterize the honey bee phage communities, we built gene-sharing networks to compare our vMAGs with those previously described bee phage studies (Fig. 2; Fig. S3) (26–29, 31), as well as reference phages taken from vConTACT3’s NCBI RefSeq database (v221). The majority of vMAGs from our analyses clustered with phage genomes described in other bee phage studies (28, 29, 31). We assigned phage taxonomy to the family level in only 5.7% (four) of our 70 vMAGs. These vMAGs were identified as *Peduoviridae*. Thirty percent of vMAGs (21) could not be identified at any taxonomic level, while all other vMAGs were identified as *Caudoviricetes*. As phages in our data set were similar to previously described phages, we investigated whether *in silico* predictions of phage host range would recapitulate earlier observations of phage host range in the honey bee gut. To perform this analysis, we utilized iPHoP (43), which aggregates multiple different prediction methods to resolve phage host prediction. Our screen resulted in 3,824 CRISPR hits between genomes found in GTDB-TK and our 70 phage genomes and culminated in 691 high-confidence host genome predictions across our phages, though many of these genomes could be resolved to the same species or genus. Corroborating earlier studies, we find that many phage genomes are predicted to target *Lactobacillus*, *Bifidobacterium*, and *Gilliamella* (26–28) (Fig. 2B). However, we identified eight phage genomes that were predicted to infect multiple microbial genomes via either hits from BLAST or CRISPR spacers. These genomes were evenly distributed between pairs of different mMAG hosts.

### Genes encoding resistance to arsenic and tetracycline are common on recovered plasmids

**Fig 3:**
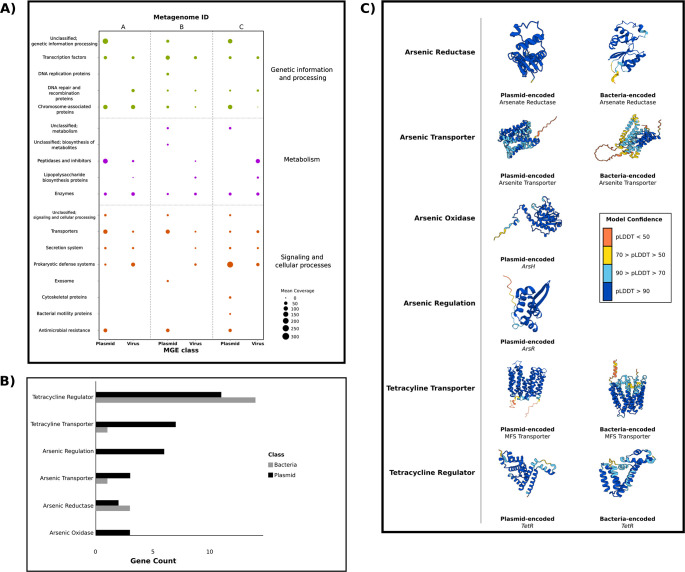
Plasmid-encoded antibiotic resistance genes are widespread in honey bees (**A**). Read coverage assigned to KEGG BRITE pathways associated with either plasmids or viruses. Larger circles correspond to higher overall coverage while colors correspond to broad functional groupings. (**B**) Gene counts for enriched gene functions (tetracycline resistance and arsenic resistance) within the honey bee gut. Bars are color-coded by host (plasmid, black; bacteria; gray). (**C**) AlphaFold3 structural predictions of representative proteins found in 3B. Protein predictions on the left column are plasmid-encoded variants, while bacterial-encoded variants are in the right column. Residues are color-coded according to pLDDT scores, indicating model confidence.

Our next goal was to understand how cargo genes, which could potentially affect microbial host fitness and evolution, were distributed among different MGEs. To identify such cargo genes, we clustered a total of 2,228 and 655 genes on vMAGs and pMAGs, respectively, into 18 protein clusters using KEGG BRITE KOs. These protein clusters can be broadly grouped into functions related to genetic information and processing, metabolism, and cellular signaling, overall representing a high degree of functional diversity. While both classes of MGEs were associated with a wide suite of systems relevant to their transmission and persistence (such as toxin-antitoxin systems, methyltransferases, and restriction-modification avoidance), we focused our analyses on MGE-encoded auxiliary metabolic genes (AMGs) (Fig. 3A). Out of 591 recovered AMGs, 237 were associated with pMAGs, representing 36.2% of the total pMAG gene content. Antimicrobial resistance genes (ARGs) were especially common and were recovered from 40.7% of all pMAGs in this study and from all individual metagenomes. These genes encoded functions related to resistance to tetracycline, chloramphenicol, and streptomycin, although genes encoding resistance to tetracycline were especially widespread on pMAGs, appearing on several individual pMAGs in each metagenome. Tetracycline resistance genes harbored similar organizational structure in each pMAG, encoding two *TetR* transcriptional regulators and a single MFS transporter (KF18476, K19047, and K08151).

In contrast to pMAGs, vMAGs were associated with far fewer AMGs, encoding 111 AMGs which represented 4.98% of the total genic diversity found in recovered phages. These viral AMGs are annotated to be involved in processes, such as iron efflux (K13283), fructoselysine conversion (K19510), and domains associated with chlorhexidine efflux transporters (PF05232.16), which are part of a recently described set of antimicrobial efflux pumps known as PACE proteins (44). Interestingly, genes associated with lipopolysaccharide biosynthesis were found exclusively on vMAGs recovered in this study, suggesting that some of these phages may modify host LPS to potentially provide resistance to co-infecting phages or help enable phage infection via LPS modification. Additionally, vMAGs encoded six hypothetical chitinases (K03791), which were distributed among five unique vMAG genomes. A full list of MGE-associated annotations and their AMGs is provided as supplemental data (Table S2).

Across MGE-encoded AMGs, the most common functions were related to mechanisms of tetracycline and arsenic resistance. We therefore next explored how these two classes of AMGs were distributed among mMAGs and pMAGs (these AMGs were absent on all vMAGs). We found that both pMAGs and all recovered mMAGs encoded a similar number of proteins known to regulate tetracycline transcription (Fig. 3B). However, mMAG genomes in our data set were nearly depleted of any mechanism that would confer tetracycline resistance. In contrast, TetR/AcrR and MFS transporters were common on pMAGs, suggesting that mechanisms of tetracycline resistance are more widely distributed on pMAGs relative to microbial hosts. This is supported by earlier work on tetracycline resistance in honey bees, where nearly identical tetracycline resistance genes were recovered from both Gram-negative and Gram-positive bacteria (32, 45) and were found to be commonly associated with MGEs (32) rather than with microbial chromosomes. The majority of genes relevant to arsenic resistance were also found on pMAGs, highlighting the importance of these MGEs in maintaining and distributing AMGs across microbial hosts. Genes relevant to both arsenic regulation and arsenic oxidation (*ArsH*) were found exclusively on pMAGs. The presence of *ArsH* on pMAGs is especially interesting, as the oxidation of arsenate to arsenite reduces ROS-mediated stress to both prokaryotic and eukaryotic cells (46). Genes encoding proteins related to arsenic reduction were found at relatively equal frequency in

both pMAGs and mMAGs, while genes related to arsenite transport were primarily found on pMAGs. High levels of arsenic contamination have been found in bee colonies across Europe and the United States (47, 48), likely due to the collection of heavy metal-contaminated pollen. To confirm that AMGs related to both arsenic resistance and tetracycline resistance can encode canonical, structurally complete proteins, we predicted their associated protein structures via AlphaFold3 (49), selecting one protein from each functional group. Overall, the majority of residues across all models exhibited pLDDT scores higher than 70, indicating high confidence regarding the predicted structure relative to the true protein structure (Fig. 3C).

### Hi-C-based linkage of MGEs and honey bee microbes identifies both plasmids and phages that interact with multiple, phylogenetically distinct genera

Having established that ecologically relevant genes are common on MGEs in the honey bee microbiome, we next investigated how these MGEs were partitioned across microbial hosts. Describing the distribution of these MGEs would help identify whether any taxa acted as major reservoirs for MGEs and their encoded functions, or if these MGEs were equally distributed among all taxa. Assigning microbial hosts to MGEs is challenging due to the heterogeneity and bias intrinsic to isolate-based approaches. Alternatively, *in silico* methods for assigning host–MGE interactions are limited, especially with respect to plasmids. To assign viral hosts, previously published approaches have relied on matching CRISPR spacers, which are encoded in assembled microbial MAGs, to protospacers found in recovered MGEs. However, many bacteria do not use CRISPR systems (50), and the repetitive nature of CRISPR arrays makes their assembly from short-read metagenomic challenging. Additionally, CRISPR spacer content reflects the history of past interactions between MGEs and hosts, not a contemporaneous snapshot. Finally, naturally recovered host–virus pairs have been increasingly shown to exhibit spatial and temporal variation (51, 52) in host range and fitness effects. This surprising heterogeneity has been observed between host bacterial populations (52), species (13), and even among viral variants (51). Within host-associated microbiomes, variation in viral host range is unlikely to be captured through the identification of historical interactions through spacer-to-protospacer matching via short-read metagenomic sequencing.

**Fig 4:**
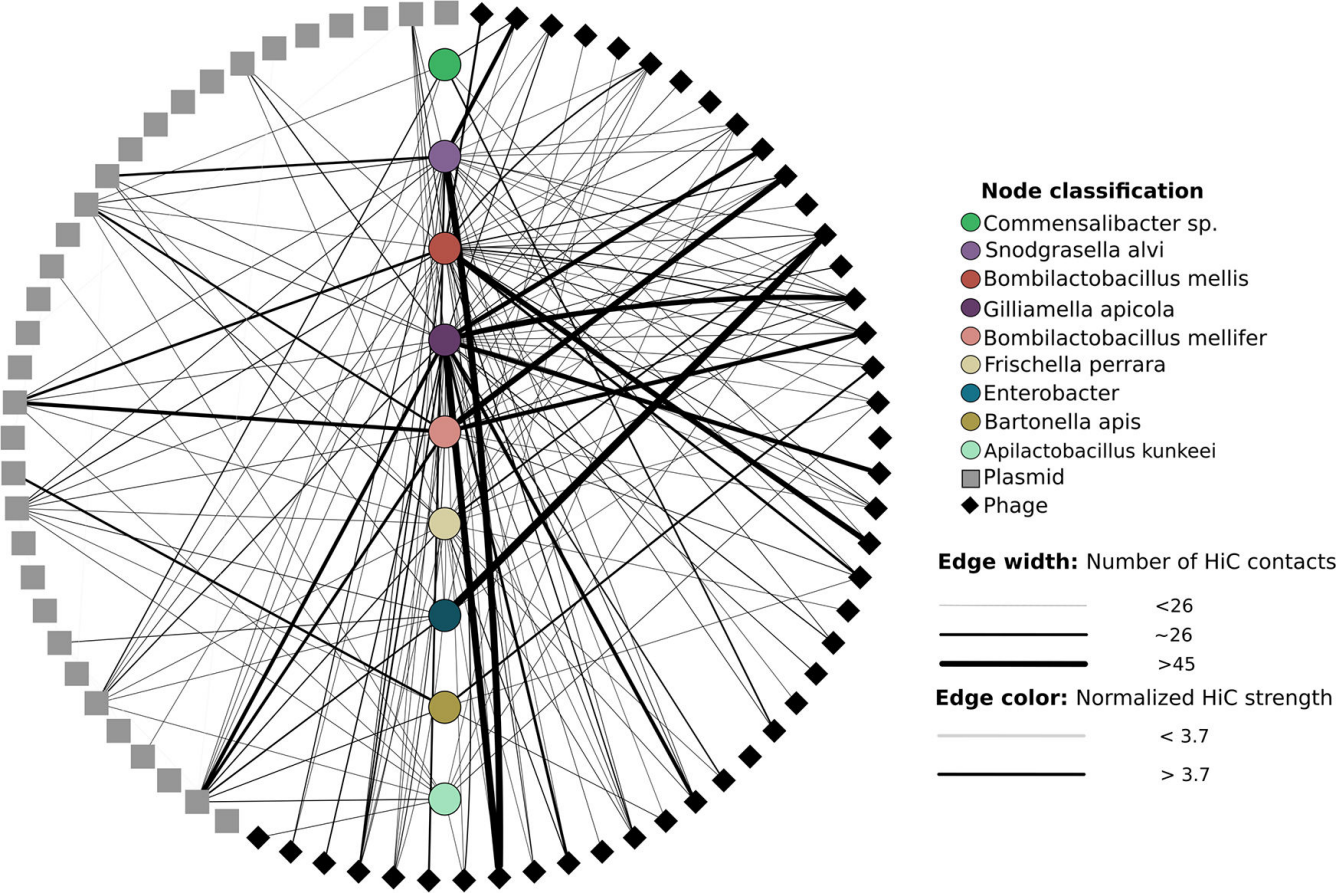
Cytoscape visualization of normalized Hi-C contacts between mMAGs, pMAGs, and vMAGs found in all metagenomes. mMAGs are centered in the circle and are color-coded by taxonomy. vMAGs are represented as black diamonds, and pMAGs are represented as gray squares. Edge width corresponds to the number of contig-to-contig linkages between either vMAGs or pMAGs and mMAGs. The darkness of each edge corresponds to the cumulative strength of normalized Hi-C contacts between two node pairs.

To address these challenges, we sequenced three Hi-C metagenomic libraries via the Phase Genomics ProxiMeta platform. After filtering, we recovered a total of 345,687,698 Hi-C paired reads (104,694,834; 79,790,796; and 161,202,068 for metagenomes A, B, and C, respectively). By mapping Hi-C reads to assembled mMAGs, pMAGs, and vMAGs, we can infer putative chromosomal interactions between microbial genomes and intracellular MGEs (see contact heat maps for each individual sample in Fig. S4-S6). We estimated noise-to-signal ratios of raw Hi-C contacts using the ratio of intra-mMAG to inter-mMAG contacts (raw noise = 0.0343 *±* 0.00417; noise-to-signal ratios were calculated as (13); see Methods for calculations of ratios). Due to the possibility of horizontal gene transfer between MGEs (vMAG-to-vMAG, pMAG-to-pMAG, and vMAG-to-pMAG), we restricted our analysis to include only contacts between mMAGs and MGEs (vMAGs or pMAGs). In the raw composite Hi-C network, we recovered 582,844 mMAG × MGE contacts (Fig. S7), while the number of mMAG × MGE contacts was reduced to 56,780 after normalization with HiCzin. We observed a high amount of variation in both the number of interacting contigs between any mMAG × MGE pair (visualized as thicker edges) and in the linkage strength (cumulative count of normalized contacts between mMAGs and MGEs; darker edges correspond to higher normalized linkage) (Fig. 4). Due to the wide variation in both the number of Hi-C connections connecting any mMAG × MGE pair and linkage strength, we calculated the average edge weight of Hi-C links between *Apilactobacillus kunkeii* and MGEs from each metagenome to use as the lower bound for which Hi-C links to include in our analysis. While *A. kunkeii* is widely distributed among honey bee larvae and the queen (30), its distribution and abundance in the worker midgut and hindgut are negligible, and we reasoned that many MGE × *A. kunkeii* contacts represent putatively spurious and weak interactions. Our results support this reasoning as *A. kunkeii* was the only mMAG that consistently aggregated both the weakest and fewest number of mMAG × MGE Hi-C links in both the composite and the individual networks (Fig. S8–S10). After filtering using this threshold, we saw a reduction of 7.85% in observed mMAG × MGE linkages for a total of 52,320 linkages in the composite network (Fig. S11). Because the Hi-C reads were mapped to a database built from all assembled high-quality mMAGs, pMAGs, and vMAGs, we performed a final filtering step to control for spurious contacts between highly similar genes shared by MGEs across different metagenomes. Within each metagenome, we removed all mMAG × pMAG contacts from pMAGs that were not originally assembled from the same metagenome. The final composite network resulted in 9,289 high-confidence mMAG × MGE interactions.

We interpret darker, wider edges between mMAG × MGE pairs as indicative of the presence of intracellular associations between the pair, though we note that the presence of linkages between any mMAG × MGE pair is not proof of *in situ* association, nor is it proof of higher rates of infection within microbial populations. Rather, we use these signals to describe the distribution of MGE interactions across microbial hosts. Overall, *Snodgrasella*, *Giliamella*, and *Bombilactobacillus mellis* aggregated the highest average number of mMAG × vMAG interactions (Fig. S12). We identified consistent predictions of phage hosts from iPHoP and Hi-C-based methods, though there was

no overlap in host range between *in silico*-based predictions and Hi-C for phages predicted to target multiple microbial hosts. However, nearly all *in silico* host predictions are corroborated by Hi-C-based methods in the raw Hi-C network, suggesting that future work would benefit from deeper optimization of how Hi-C linkages are filtered. Furthermore, we note that both *Bifidobacterium* and *Lactobacillus* are not present in our Hi-C network, which prevents comparisons of host prediction between iPHoP and Hi-C-based methods in these taxa. 51.29% of mMAG × MGE linkages (4,764) were made up of mMAG × vMAG interactions, while the remaining linkages (4,525) consisted of interactions between mMAGs and pMAGs. The viral linkages could be dereplicated down to 95 unique links shared between 9 mMAGs and 42 vMAGs, while the pMAG × vMAG linkages could be dereplicated down to 53 unique links shared between 9 mMAGs and 12 pMAGs (Fig. 2; individual metagenome networks are shown in Fig. S8-S10).

### Plasmid networks are highly individualized and exhibit broad host range variation

**Fig 5:**
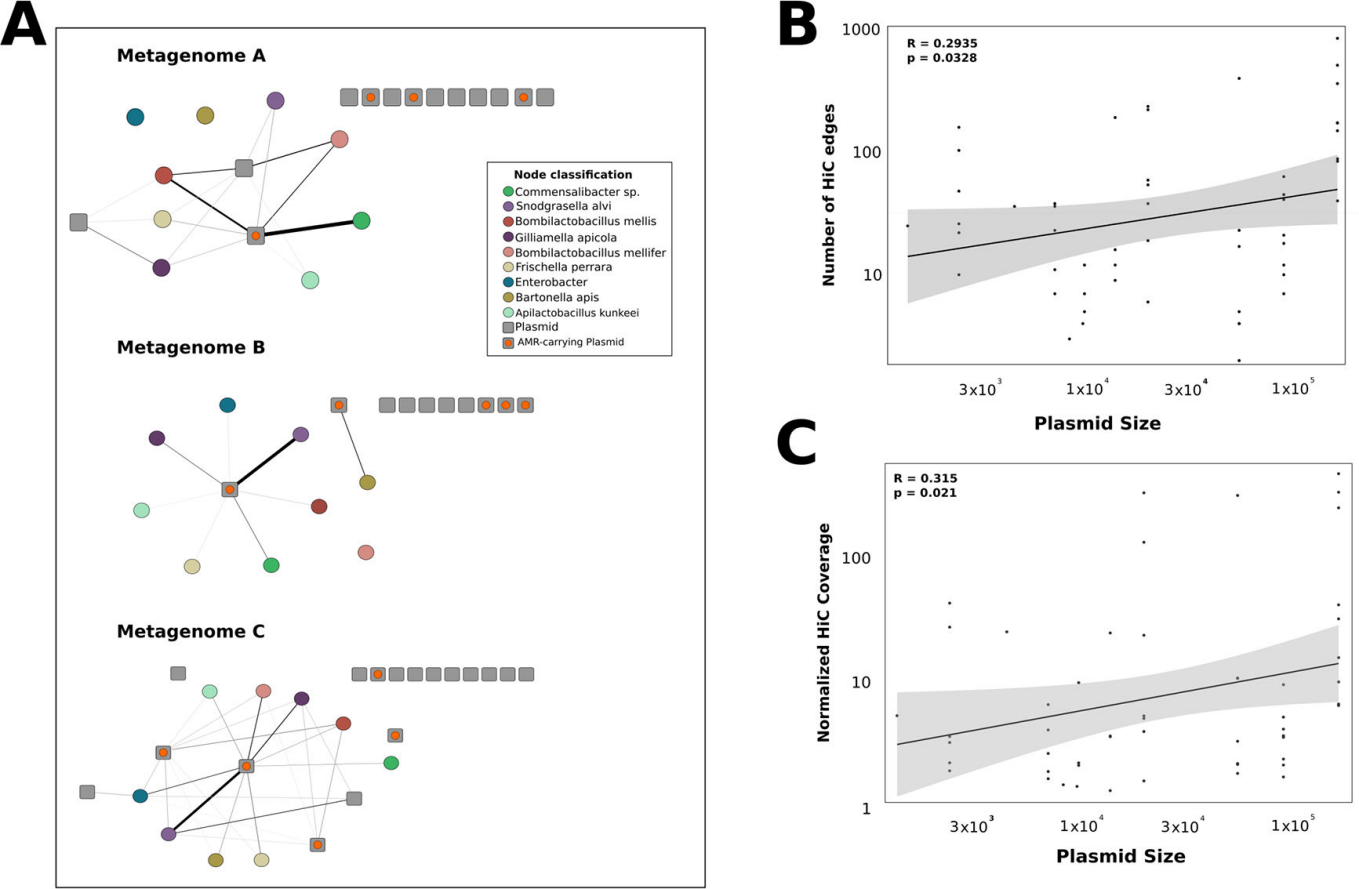
Large plasmids are distributed evenly among microbial hosts. (**A**) Individual plasmid networks for each metagenome. Networks are built from significant Hi-C connections between mMAGs (circles, colored by taxonomy) and pMAGs (gray square). pMAGs with orange dots indicate pMAGs associated with antibiotic resistance genes. As in Fig. 4, edge width corresponds to the number of contig-to-contig linkages between pMAGs and mMAGs. The darkness of each edge corresponds to the cumulative strength of normalized Hi-C contacts between two node pairs. (**B**) Counts of the number of unique pMAG interactions aggregated across microbial hosts within each metagenome. The number of interactions is on the X-axis; the taxonomy of the microbial host is on the Y-axis. (**C**) Relationship between plasmid size and the number of Hi-C edges (number of contig-to-contig interactions between mMAGs and pMAGs). Rho and *P-values* generated from a Spearman’s rank correlation.

Next, we wanted to explore whether individual plasmidomes exhibited significant differences in patterns of host range variation. To investigate this, we created individualized plasmid networks from filtered Hi-C contacts to compare how individual plasmids are partitioned across microbial hosts in each microbiome (Fig. 5A). We found that the majority of plasmids within each metagenome showed no significant Hi-C-based association with any microbial host. We do not interpret this to mean that these plasmids lack hosts. Rather, we interpret the absence of host–plasmid connections in each network to indicate that these plasmids exhibit relatively weak interactions with their microbial hosts, likely due to low frequency and/or copy number within microbial populations. Alternatively, these plasmids may be associated with an alternative microbial host that did not pass our genome quality thresholds. In all metagenomes, the distribution of unique, significant Hi-C pMAG interactions was fairly homogeneous across the available microbial hosts (Fig. 5B). However, we note that *Gilliamella*, *B. mellis*, and *Frischella* aggregated the most interactions in both metagenomes A and C. In each metagenome, plasmids with significant interactions were evenly dispersed across the recovered microbial genomes. However, the networks were fairly sparse, with only 13.6%, 8.1%, and 18.4% of the maximum number of pMAG × mMAG interactions in metagenomes A, B, and C, respectively. Within each metagenome, plasmids exhibited substantial variation in host range. Within metagenome A, significant interactions were recovered between pMAGs AD311, AC411, and AA192, which interacted with 3, 7, and 6 hosts, respectively. In metagenome B, only two plasmids exhibited significant interactions: pMAG AB620 interacted with a single host, while pMAG AB960 exhibited seven interactions. Finally, metagenome C included seven plasmids with significant interactions. Of these, three plasmids (AB307, AG959, and AC774) interacted with a single microbial host, while the remaining plasmids interacted with 4 (pMAG N4), 5 (AD311), 8 (AF889), and 9 (AC137) microbial hosts. In all metagenomes, plasmids with the broadest host range encoded genes for antibiotic resistance. This suggests, like in many other microbial communities, that plasmids are important for the dispersal of antibiotic resistance genes across a phylogenetically broad set of hosts within the honey bee microbiome. Larger plasmids across all metagenomes exhibited significant positive correlations with the number of significant contig-to-contig linkages between mMAGs and pMAGs (Fig. 5C, *R* = 0.2935, *P* = 0.0328) and the number of filtered Hi-C reads connecting mMAG and pMAG contigs (Fig. 5D, *R* = 0.315, *P* = 0.021), suggesting that larger plasmids have broader host ranges within this system. We note biases intrinsic to Hi-C metagenome data. In particular, Hi-C signal is known to be biased toward contigs with higher coverage and larger size (53). However, linear models showed no significant effect of read depth on the number of mMAG × vMAG Hi-C linkages (*P* = 0.8065, Fig. S13) or mMAG × pMAG Hi-C linkages (*P* = 0.8846, Fig. S14) discovered herein, while larger vMAGs were associated with fewer interactions (*P* = 1.79*e^−^*^12^).

### Near-identical gene modules found on broad host range plasmids in different honey bee colonies

**Fig 6:**
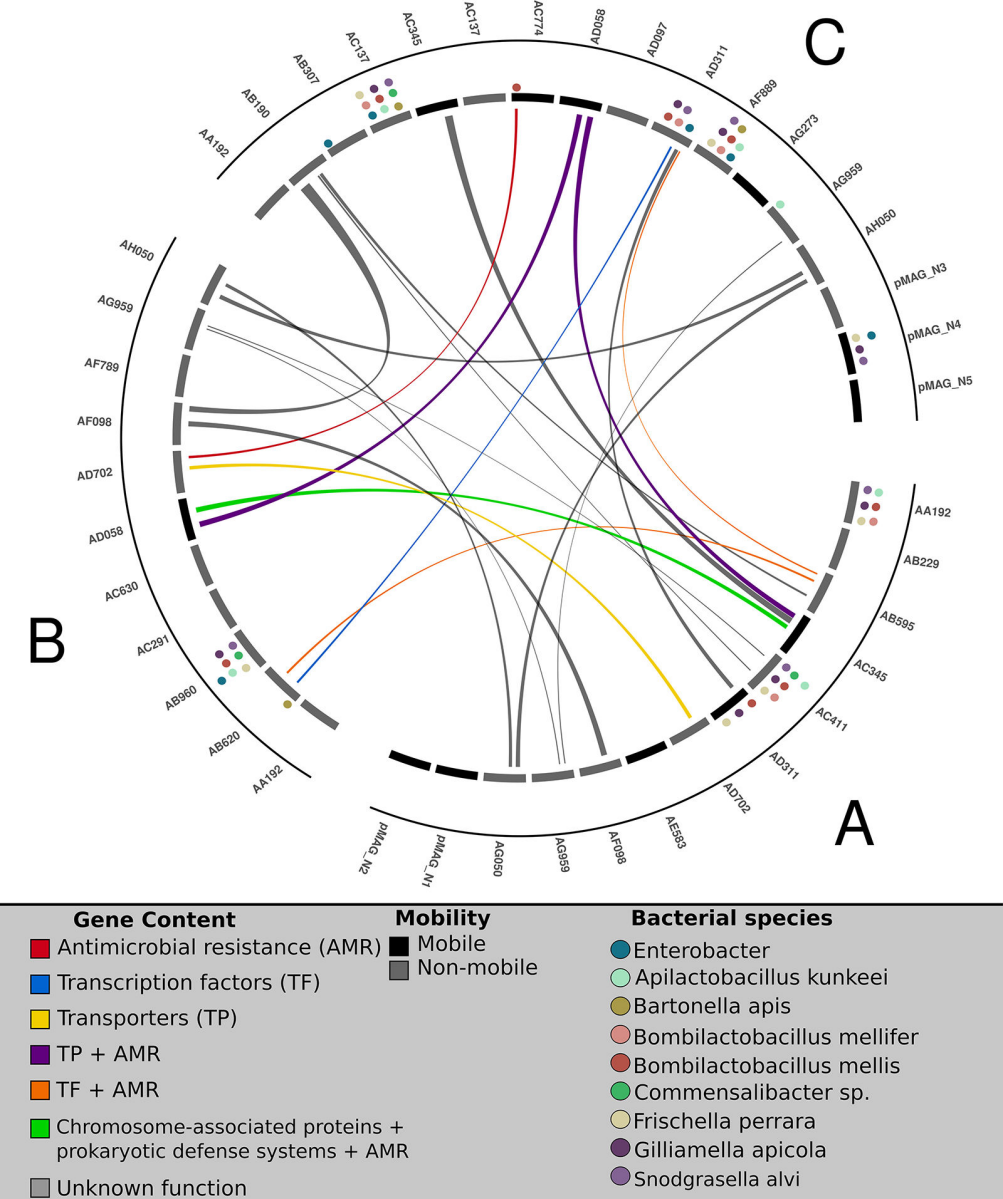
Nearly identical genes are found on plasmids that exhibit large differences in host range. Circos plot illustrating high similarity (*≥*97%) between genes found on all pMAGs recovered from each metagenome. pMAGs are demarcated by individual rectangles (inner arc) and are colored via the presence (black) or absence (gray) of genes encoding machinery for plasmid mobilization. Colored circles above each pMAG represent putative connections between pMAGs and mMAG hosts as inferred via Hi-C linkage data (Fig. 4). All pMAGs found within a single metagenome are enclosed within a single arc (outer arc). Lines connecting pMAGs represent the presence of one or more genes with high similarity on both pMAGs. Lines are color-coded by the annotations associated with highly similar genes. The names of each plasmid are provided above each single arc.

While the Hi-C networks within each individual metagenome suggest that significant interactions between microbial hosts and plasmids are fairly sparse, the presence of plasmids with identical relaxase types in multiple honey bee colonies suggests that plasmids are potentially trafficked between colonies. MGEs, such as plasmids and viruses, can serve to mediate horizontal gene transfer in microbial communities, with important genetic loci moving between MGEs and mediating functional changes in the microbial hosts. To identify genes associated with putative HGT events (i.e., mobile genes), we used a previously benchmarked approach (54, 55) based on the identification of nearly identical genes present in phylogenetically distant hosts. We used this method to identify 179 mobile genes from the three honey bee metagenomes. 63.5% of these mobile genes were found on distinct pMAGs (Table S3), while the remaining mobile genes were identified on mMAG × mMAG pairs or vMAG × vMAG pairs. 17.7% of all plasmid-associated genes exhibited *≥*97% ANI with at least one another plasmid-encoded gene, suggesting that a significant degree of genic diversity within honey bee-associated pMAGs has been recently acquired via HGT. Half of all identified pMAG-associated ARGs (21/42) were classified as putatively mobile. In addition to ARGs, we identified highly similar genes between plasmid pairs that encode functions such as transcription factors, transporters, chromosomal-associated proteins, and prokaryotic defense systems (Fig. 6). Many identified gene pairs were found on plasmids identified as broad-host-range, as inferred from our Hi-C data. For example, pMAG AC 345 in metagenome A is associated with any known detectable microbial hosts, yet harbors three putative HGT events with pMAGs recovered from the two other honey bee colonies, potentially expanding the host range of these HGTs to six other bacterial hosts. In another case, pMAG AB595 in metagenome A exhibits significant interactions with microbial hosts, yet shares nearly identical genes conferring tetracycline resistance on plasmids AB620 in metagenome B (one host) and AD311 in metagenome C (five hosts). The nested nature of these interactions suggests that while plasmids may not exhibit strong host associations within individual microbiomes, they may potentially affect microbial evolution through the distribution of important cargo genes to other host-associated MGEs within the same microbial community. Interestingly, plasmids encoding their own transmission machinery were no more likely to harbor predicted HGT events (Welch’s *t*-test, df = 11.265, *P* = 0.09).

**Fig 7:**
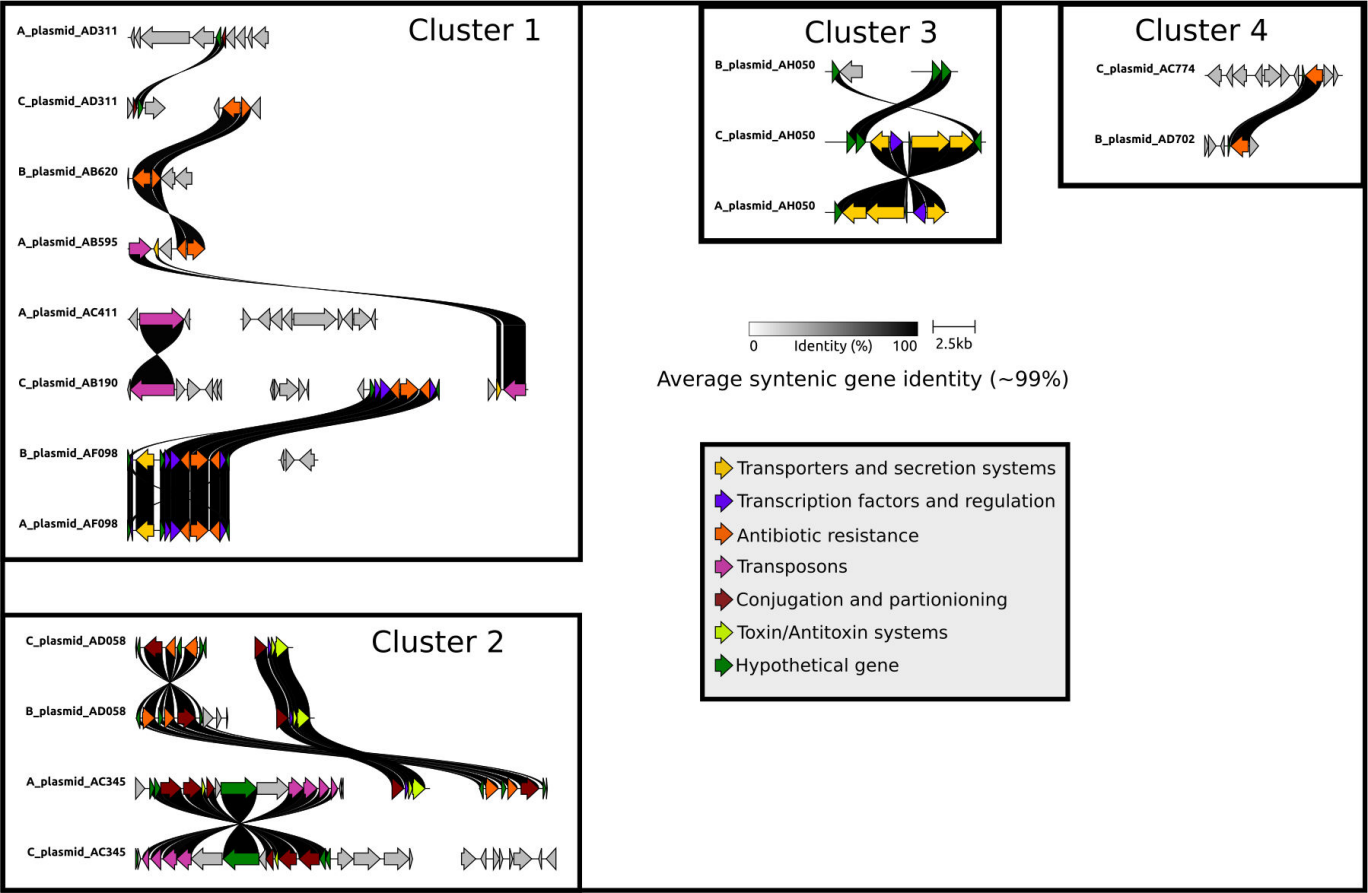
Mobilized operons maintain architecture across plasmids in different microbiomes. Gene arrow maps built from pMAGs sharing *≥*2 genes of high identity. Arrows are color-coded by predicted function. Only ribbons corresponding to *≥*99% identity shared between genes are shown. Metagenome identity for each pMAG is given as the first character in pMAG ID (i.e., A plasmid AD311 was recovered from metagenome A). Each cluster includes all pMAGs, where at least one gene shared high similarity with another pMAG-associated gene.

Our results corroborate earlier studies in the honey bee microbiome that recovered nearly identical ARGs from isolates sampled over a wide geographic range (32, 33). However, in this work, we are able to identify how these mobile genes were arranged on the plasmid backbones. This deeper understanding, including shared synteny, provides stronger evidence for gene mobilization across colonies. We identified four clusters of pMAGs containing syntenic genes exhibiting *≥*99% similarity (Fig. 7). In cluster 1, comprising 46 mobile genes, we identified near-identical genes shared across multiple plasmid types and across multiple metagenomes. For example, plasmid AB595 from metagenome A, plasmid AB620 from metagenome B, and plasmid AD311 from metagenome C all contained a nearly identical (*≥*99%) gene pair (KEGG annotations: TetR/AcrR tetracycline repressor and MFS transporter, tetracycline resistance protein). In this same cluster, plasmid AB595 from metagenome A is further connected to another pMAG in metagenome C (C plasmid AB190) via a set of paired genes encoding a transposon and phosphoryltransferase, while C plasmid AB190 encodes a large antibiotic resistance cassette, encoding multiple genes for tetracycline resistance, transcription regulation, and DNA binding. This entire antibiotic resistance cassette is nearly identical to cassettes recovered from pMAGs in the two other metagenomes (B plasmid AF098 and A plasmid AF098). Nearly identical cassettes are repeatedly observed on multiple pMAGs across all three metagenomes in all of the other clusters, including several genes encoding transmembrane secretion effectors (PF05977.17), ABC transporter domains (PF00005.31), and transcriptional regulations (PF21259.1) in cluster 3. In cluster 2, we observed several examples of AMR genes shared across multiple pMAGs in all three metagenomes. These AMR genes are flanked by several transposon genes, suggesting putative mechanisms of mobilization.

## DISCUSSION

Mobile genetic elements drive a significant amount of adaptive evolution among microbial hosts within intra-host microbial communities, either through direct co-evolutionary interactions (antagonistic co-evolution with phages) or through the acquisition of some ecologically important genes (such as plasmid-associated antibiotic resistance genes). However, interactions between MGEs and their microbial hosts are highly dynamic, and there is a severe paucity of information regarding microbe × MGE interactions *in situ*. Our study directly addresses this gap in knowledge by combining metagenomics and proximity ligation (Hi-C) methods with the highly tractable honey bee worker as a model microbiome. Using conservative thresholds for MGE calling, we show that honey bee colonies co-housed in the same apiary exhibit highly variable phage and plasmid communities within age-matched worker guts and that these communities exhibit a large degree of genic and functional diversity. However, we note that our study does not capture all of the interactions between honey bee-associated MGEs and core microbial taxa, as both *Lactobacillus* and *Bifidobacterium* MAGs were discarded to minimize bias induced from the high contamination in these genomes. We used a conservative approach to minimize false positives arising from misassemblies, and we look forward to further empirical work that explores MGE ecology and evolution within the honey bee system. Our observations of high phage diversity and *in silico* host predictions recapitulate findings from other studies that investigate honey bee-associated phage communities (26–29, 31), as well as findings from bee-associated plasmid studies (32, 33). We note that each of our metagenomic samples consists of 15 pooled workers, and our observations of MGE diversity may be driven by heterogeneity among individual worker microbiomes. The genetic background of workers has been shown to have an impact on strain-level variation within the worker microbiome (56). We attempted to control for this by using single-drone inseminated queens, age-matched workers, and by sampling hives co-housed in the same apiary, but future work is needed to characterize the community-level variation of MGEs between individual workers.

Through the use of Hi-C sequencing, we show that both viruses and plasmids likely interact with phylogenetically different microbial hosts in highly structured and extensively co-evolved communities, such as the *A. mellifera* worker microbiome.

Although broad host range plasmids were discovered nearly 50 years ago (57), few studies have explored these MGEs in the honey bee microbiome. However, Sun et al. identified highly similar ARGs from assembled contigs across most core microbial taxa in the honey bee microbiome (33). They further showed that this ARG can be successfully transmitted across *Gilliamella*, *Snodgrassella*, and *Bartonella* via conjugation by a single plasmid, supporting the observations of plasmid host range in this study. However, the breadth and variation in observed phage host range here were surprising. Our observations are corroborated by several recent studies that use both Hi-C and CRISPR-based methods to resolve interactions between microbes and their bacteriophages (13, 14, 58). Hwang et al. (13) proposed several models to explain the observation of broad taxonomic host range in bacteriophages from a deep sea microbial mat, proposing scenarios such as high density of viral particles among a dense, highly diverse microbial community; the transfer of viral particles and/or genomes among syntrophic microbes; or bona fide host switching or host range expansion in viral populations. While our observations of genic variation among variants of the same viral species, with respect to differences in viral host range, might support the latter model of viral host switching or range expansion, we stress the need for further experimental work to validate the ability of virions to associate with phylogenetically distant hosts. We emphasize that our observations of vMAG interactions with multiple mMAG hosts are not claims of successful phage infection and reproduction in these hosts. Many of these interactions could result in aborted infections or failure in replication. We also emphasize that our vMAGs represent populations of highly similar phage genomes (5% ANI). Genetic tools developed to study phage host range have shown that only a small number of mutations are required in the tail gene *gp17* to modify phage host range from *E. coli* to *Klebsiella* or *Yersinia* (59). Multiple phage subpopulations within our vMAGs, each persisting on a different microbial host, could potentially explain the observed variation in host range.

The observed host range variability of MGEs is likely an important factor in microbial evolution and ecology due to the high degree of MGE-encoded functional and genetic diversity. Indeed, while plasmids exhibited wide variation in host range, we found that over 17% of plasmid-encoded genes (and over 50% of ARGs) were nearly identical (*≥*97%) to a gene on another plasmid. The high similarity of these genes across plasmids associated with different honey bee colonies may be due to the recent, independent introductions of these genes into honey bee colonies and/or strong-selective pressures within each individual colony. However, the conservation of gene synteny between plasmid pairs found in different colonies supports recent plasmid dispersal and subsequent recombination as opposed to environmental selection on single genes. These findings corroborate earlier studies in the honey bee plasmid literature, which identified nearly identical ARGs from plasmid-like sequences found in geographically disparate honey bee colonies (32, 33). The genes identified in these studies were related to tetracycline resistance, which we also identify in our work. The prevalence of near-identical tetracycline resistance in these studies (and our own) is likely driven by five decades of tetracycline usage for larval foulbrood control in honey bees. Understanding which mechanisms drive local adaptation within honey bee colonies—especially colonies proximal to one another—remains an open question (54). As many of the observed plasmid gene pairs may be transient, either due to neutral or deleterious fitness effects, future work should consider the impact of temporal and spatial variability on mobile gene dynamics, as patterns of mobile gene acquisition and stable co-occurrence may emerge from densely sampled colonies.

Interactions among mobile genetic elements and microbial hosts drive many fundamental evolutionary and ecological processes within host microbiomes. Developing a deeper understanding of these interactions is crucial if we are to generate testable hypotheses regarding the response of host-associated microbial communities to perturbation or disturbance, such as in the case of antibiotic usage. Our work highlights the variability of MGE × microbe interactions within host-associated microbial communities and suggests a deeper complexity in these systems than previously thought. Beyond the transfer of genes across wide phylogenetic distances and the driving of adaptive responses in microbial hosts, theory predicts that variation in HGT rates among plasmids affects plasmid gene gain and loss (60). With respect to phages, many hypotheses that explore phage population dynamics, such as kill-the-winner or evolutionary arms races, often consider the co-evolution of a phage alongside a single bacterial host species. While we await further empirical validation of phage host range variation (61), the observations of this study (and others [13, 14, 57]) suggest that bacteriophages may be engaging in antagonistic co-evolution among multiple bacterial species within a community. Future ecological and evolutionary work may benefit from incorporating these observations into predictive models.

## MATERIALS AND METHODS

### Honey bee management, metagenomic sequencing, and Hi-C sequencing

Honey bee samples were collected from managed single-drone inseminated (SDI) colonies at the Bee Research Facility at the University of Illinois at Urbana-Champaign. SDIs were chosen to minimize intra-colony variation among workers (62). Each colony was located within the same apiary in order to minimize variability associated due to changes in distance and acquired environmental resources. For each metagenomic sample, a frame was pulled from the colony and visually inspected for the queen.

Workers were quickly brushed from the frame and moved to 4C until bees were immobilized. For each individual colony, 15 age-matched workers were removed and dissected, retaining the entire gut of the worker. These guts were pooled and transferred to a sterile dounce homogenizer. Three milliliters of cold PBS were added to each homogenizer, and the bee tissue was homogenized on ice. Three milliliters of homogenate were then pipetted into three sterile 1.5 mL microcentrifuge tubes and spun down at 500 × *g* for 10 minutes to pellet host tissue. Supernatant from all three microcentrifuge tubes was transferred to a sterile Falcon tube. 1.5 mL of the pooled supernatant was then transferred to a new microcentrifuge tube and spun down at 5,000 × *g* for 10 minutes. Following this last centrifuging step, the supernatant was removed, and the bacterial pellet was flash-frozen with liquid nitrogen before being stored at −80°C. This process was repeated for all metagenomic samples. All samples were sent to Phase Genomics for DNA extraction and the generation of short-read and Hi-C proximity ligation libraries via Phase Genomics’ ProxiMeta service. gDNA used for metagenomic sequencing was extracted using zymoBIOMICS DNA Mini Kit. Metagenomic libraries were prepared using a HyperPrep kit (KAPA Biosystems) as referenced in (63). Hi-C libraries were prepared from a ProxiMeta Hi-C kit. All kits were used according to the manufacturer’s instructions. Hi-C libraries were prepared using the restriction enzymes, Sau3AI and MluCI. Both Hi-C and 150 bp short-read libraries were sequenced on a single lane of an Illumina NovaSeq.

### mMAG assembly, binning, and annotation

Reads from the metagenomic library were filtered by quality score and trimmed using BBduk (BBMap - Bushnell B. - sourceforge.net/projects/bbmap/) before assembly with MEGAHIT (64) (–min-contig- len 1000 –k-min 21 –k-max 141 –k-step 12 –merge-level 20,0.95). Microbial metagenomically assembled genomes (mMAGs) were binned by combining results from multiple binning software, including Maxbin2 (65), MetaBAT2 (66), and HiCBin (67). These bins were used as input for DASTool (68). mMAG quality was evaluated using checkM (69). We retained only medium- to high-quality mMAGs (*≥*50% completeness and *≤*10% contamination) from each metagenome. mMAGs from each metagenome were dereplicated at 97% average nucleotide identity (ANI) using dREP (70). mMAGs were taxonomically assigned using GTDB-Tk (71) on KBase (72). Dereplicated mMAGs that were representative of the known phylogenetic diversity within honey bee workers were used for all further analyses. mMAG genes were predicted using Prokka (73) and annotated using DRAM (74) and METABOLIC (75) using default databases (DRAM-setup.py prepare databases –output dir DRAM data –skip uniref).

### vMAG binning and annotation

Contigs from each metagenome and *≥*1,000 bp in length were predicted as viral using VIBRANT v1.2.0 (76). Contigs predicted as viral by VIBRANT were used as input for CheckV (38). Viral contigs that were classified as medium to high quality by CheckV were retained for all further analyses. Retained viral contigs were dereplicated with CD-HIT (77) at 95% ANI and 85% breadth (cd-hit-est -i $cat file-o $cat file replicate -c 0.95 n 10 -aS 0.85). Dereplicated viral contigs (vMAGs) were taxonomically identified using vConTACT3 (https://bitbucket.org/MAVERICLab/vcontact3/src/master/). vMAGs were annotated using Cenote-Taker2 (78) and DRAM-V and were screened for putative auxiliary metabolic genes (AMGs) using both VIBRANT and DRAM-V.

### pMAG binning and annotation

Plasmid contigs from each metagenome were predicted using MOB-recon, a subprogram of MOB-suite (37). Contigs *≥*1,000 bp in length were given as input for MOB-recon. Predicted plasmids were then clustered within each metagenome at 95% ANI and 85% breadth using CD-HIT. Due to the difficulty in assembling plasmids from short-read metagenomic data sets, all plasmid bins were checked for contigs that had assembled into both mMAG and plasmid bins. These contigs were excluded from the plasmid bins. Finally, all plasmid bins from all metagenomes were dereplicated at 95% ANI and 85% breadth to enable comparison across metagenomes. These dereplicated plasmid bins (pMAGs) were annotated using DRAM and METABOLIC. Because DRAM and DRAM-V expect either microbial or viral contigs, we could not utilize AMG scoring for pMAGs. Instead, pMAGs were manually inspected for the presence or absence of AMGs via annotations derived from KEGG (79) and PFAM (80).

### Hi-C-based reconstruction and normalization of mMAG and MGE associations

Hi-C reconstruction of mMAGs and MGEs closely followed the methods described by Hwang et al. (13). Hi-C reads were quality-filtered using BBduk and mapped using bwa mem (bwa mem −5SP) (81) against a combined database containing all mMAGs, pMAGs, and vMAGs. All contigs in this database were dereplicated a final time using CD-HIT at 95% ANI and 85% breadth to minimize Hi-C reads mapping across highly similar contigs. Metagenomic short reads from each individual metagenome were also mapped against this database, and read coverage was calculated using bbduk. Hi-C contact maps were normalized using the unlabeled version of HiCZin (67) (hiczin.py norm -e Sau3AI -e MluCI). Normalized contacts were further filtered by removing all contacts with lower contact strength (e.g., Hi-C paired read coverage) than the average contact strength observed between *Apilactobacillus kunkeei* and *A. kunkeei*-associated MGEs. Hi-C-based contacts between mMAGs and MGEs were visualized using Cytoscape 3.10.2 (82).

### Inference of horizontal gene transfer between mMAGs and MGEs

To identify putative horizontal gene transfer events, we used a method benchmarked by Smilie et al. and Brito et al. (54). All contigs in the deduplicated database described in the previous section (Hi-C-based reconstruction of mMAG and MGE association) were screened in a pairwise manner with BLAST (blastn -query $f -db $db -evalue 1e-6 -perc identity 97 -outfmt ’10’). Previous approaches used an identity threshold of *≥*99% sequence identity with sequences *≥*500 bp in length between any two distantly related genomes (54). We relaxed this threshold to *≥*97%, as previous work (83) found that 16S rRNA gene sequences from distinct core phylotypes in the honey bee microbiome cluster at *≥*97% sequence identity. We note that the identification of highly identical sequences using this method does not imply direct transfer between any plasmids.

### Statistical analyses

All statistical analyses were performed using R v4.4.1. Prior to building linear regressions and linear mixed models, all data was standardized using the Box-Cox transformation in order to ensure the normality of residuals. All linear regression analyses and *t*-tests were built using the *R* function, ”summary.lm()” (84).

### Noise-to-signal calculations

Raw noise-to-signal ratios were calculated as Hwang et al. (13)


$$\frac{Number\;of\;inter\;-\;mMAG\;HiC\;contacts}{Number\;of\;intra\;-\;mMAG\;HiC\;contacts}$$

where *inter-mMAG contacts* are equal to the number of Hi-C read pairs mapping to different mMAGs and the number of *intra-mMAG contacts* is equal to the number of Hi-C read pairs mapping to the same mMAG.

### Effect of read depth, contig length, and MGE size on Hi-C networks

The effect of read depth and MGE size on the number of mMAG × MGEs Hi-C linkages was tested via multiple linear regressions:

*Y* ∼ Read depth

*Y* ∼ MGE size

*Y* ∼ Contig length

where *Y* represents either the number of HiC contacts or the unique number of microbial hosts for either vMAGs or pMAGs. The significance of these multiple linear regressions was evaluated with the *t*-test of the R function *summary.lm()*. The *p.adjust()* function of the R package “stats” was used to adjust the linear regression *P*-values using FDR correction.

## Data Availability

Raw metagenomic and Hi-C reads are available from NCBI under BioProject PRJNA1206451. Links to many of the Python workflows, as well as greater detail regarding how our analysesanalyzes were done, can be found here: (https://github.com/en-nui/HoneyBeeHiC_public)

## SUPPLEMENTAL MATERIAL

Supplemental material mbio.02243-25-s0001.pdf Fig. S1-S14 and supplemental table captions.

Table S1 mbio.02243-25-s0002.csv Average coverage for each mMAG contig across each metagenome.

Table S2 mbio.02243-25-s0003.xlsx Full list of MGE-associated annotations and their AMGs.

Table S3 mbio.02243-25-s0004.csv All putatively mobile genes that were recovered from this study.

ASM does not own the copyrights to Supplemental Material that may be linked to, or accessed through, an article. The authors have granted ASM a non-exclusive, world-wide license to publish the Supplemental Material files. Please contact the corresponding author directly for reuse.
