# Supplementary material for: Hi-C-resolved metagenomics reveals host range variation among mobile genetic elements within the European honey bee
Source: mBio. 2025 Sep 22;16(11):e02243-25. doi: 10.1128/mbio.02243-25 (PMC12607879; doi:10.1128/mbio.02243-25)
Supplement: Supplemental material — Fig. S1-S14 and supplemental table captions. [file mbio.02243-25-s0001.pdf]

Supplementary Information for

**HiC-resolved metagenomics reveals host range variation among mobile genetic elements within the European Honey bee**

Chris R. P. Robinson[a], Adam G. Dolezal[b], Ivan Liachko[c], Irene L. G. Newton[a]

[a]Department of Biology, Indiana University, Bloomington, IN, 47404, United States

[b]University of Illinois Urbana-Champaign, Urbana, IL 61801, United States

[c]Phase Genomics, 1617 8th Ave N, Seattle, WA 98109, United States

Corresponding authors: Chris R. P. Robinson, Irene L. G. Newton

Email: robinch@iu.edu; irene.newton@gmail.com

**This PDF file includes:**

Legends for Supplemental Tables S1 to S3

Supplemental Figures and legends S1 to S14

References for supplemental material

# Legends for Supplemental Tables S1 to S3

**Supplemental Table 1:** Read depth, contig lengths, and taxonomy information for all bacterial contigs that were used in this study.

**Supplemental Table 2:** Table containing all annotations and AMGs that were recovered from metagenomically-assembled MGEs.

**Supplemental Table 3:** Table containing all putatively mobile genes that were recovered from this study. Genes listed share ≥97% nucleotide identity with at least one other gene from another metagenome. Source and target associations for each gene are provided as well as the gene annotation.

## Supplemental Figures S1 to S14

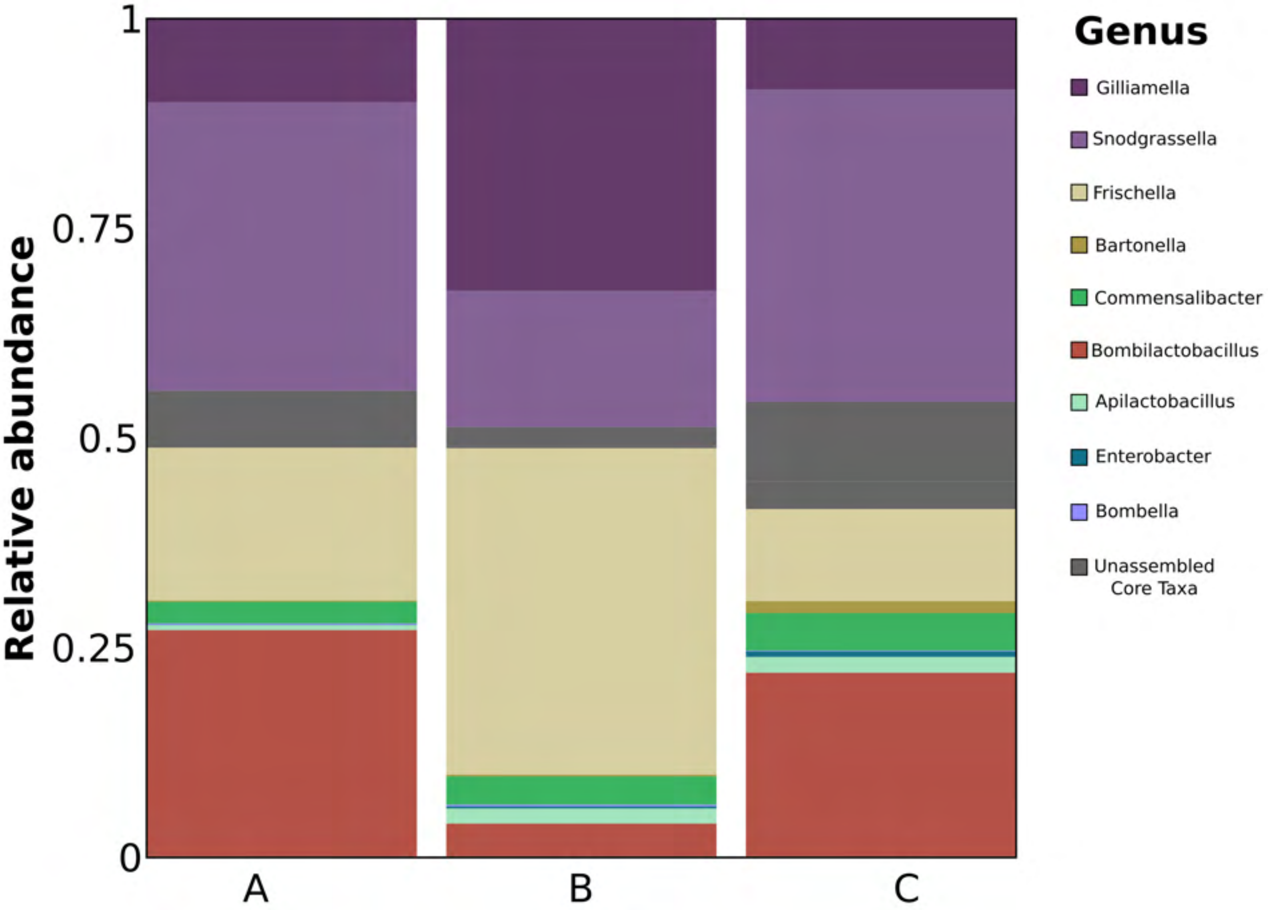


Supplemental Figure 1: 16S rRNA gene-based relative abundance of core and common microbial genera within the honey bee worker microbiome. Individual taxa are colored by genus. Taxa without high-quality assembled genomes are colored in dark gray.

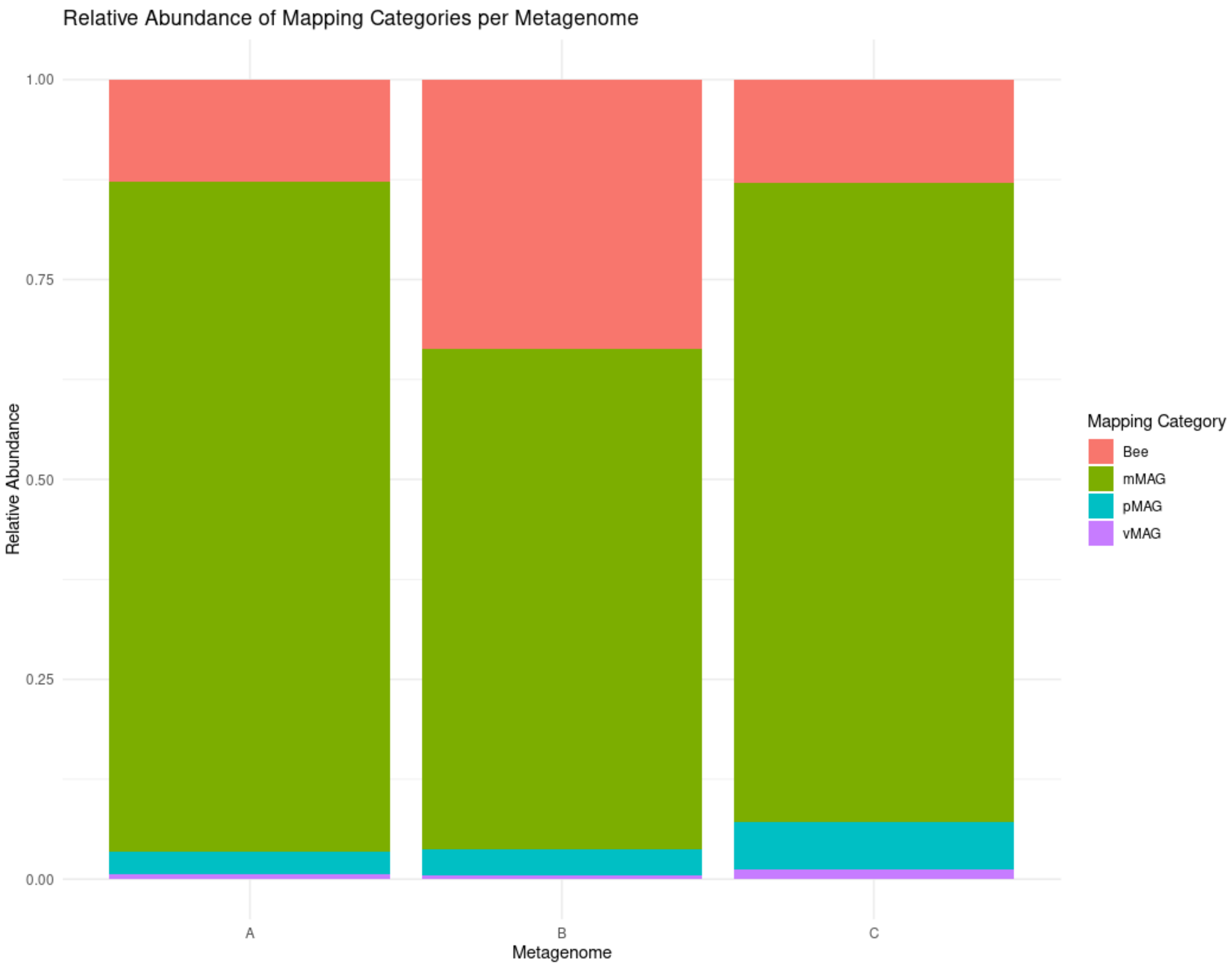


Supplemental Figure 2: Relative abundance of metagenomic reads mapping to either the honey bee genome (Pink), assembled mMAGs (Green), assembled vMAGs (purple), or assembled pMAGs (blue).

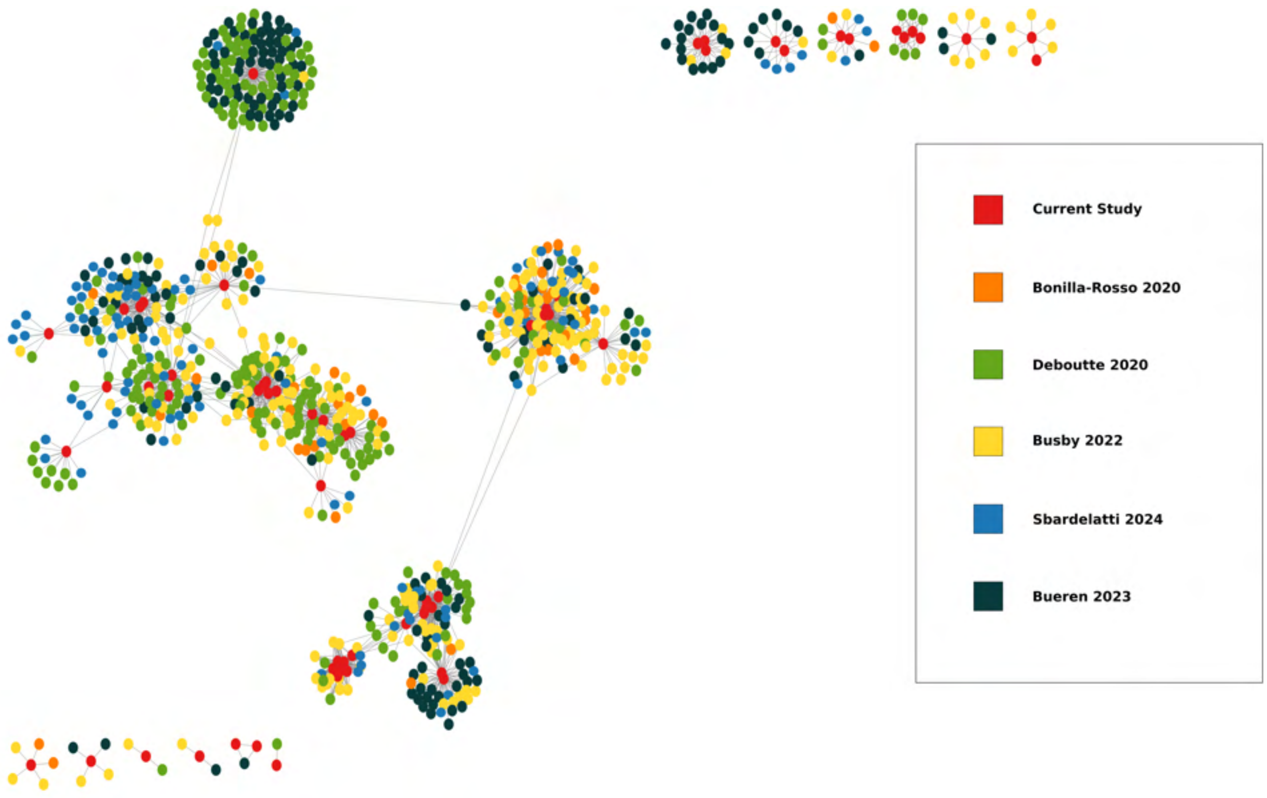


Supplemental Figure 3: vConTACT3 network generated from phage genomes recovered from this study (red) and other phage studies in the honey bee worker metagenome (all other colors).

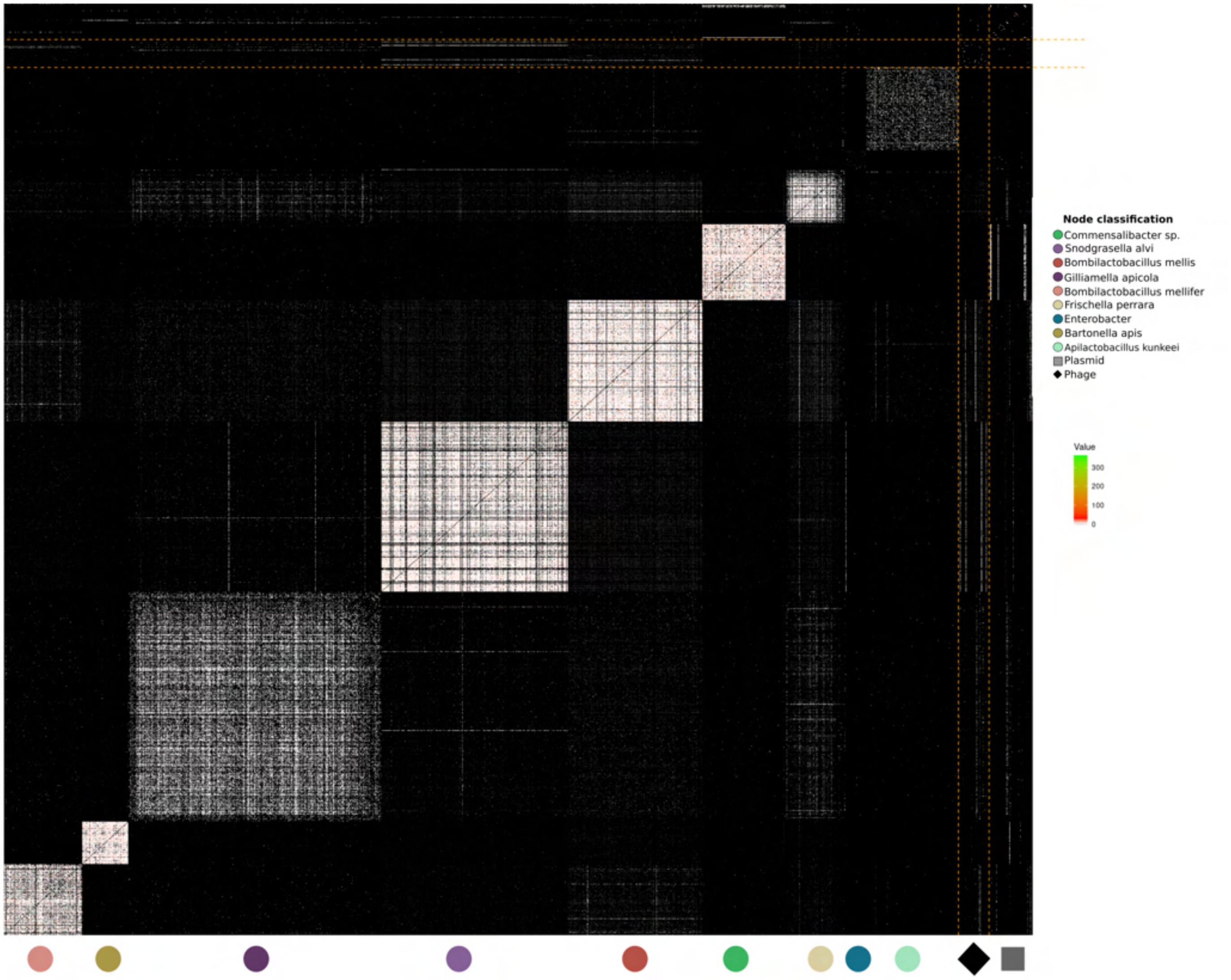


Supplemental Figure 4: Normalized HiC heatmap for Metagenome A. Contig-to-contig interactions with coverage $\geq 1$ are shown in white with more significant interactions appearing red and green. Contigs corresponding mMAG assemblies, vMAG assemblies, and pMAG assemblies are shown on the X-axis.

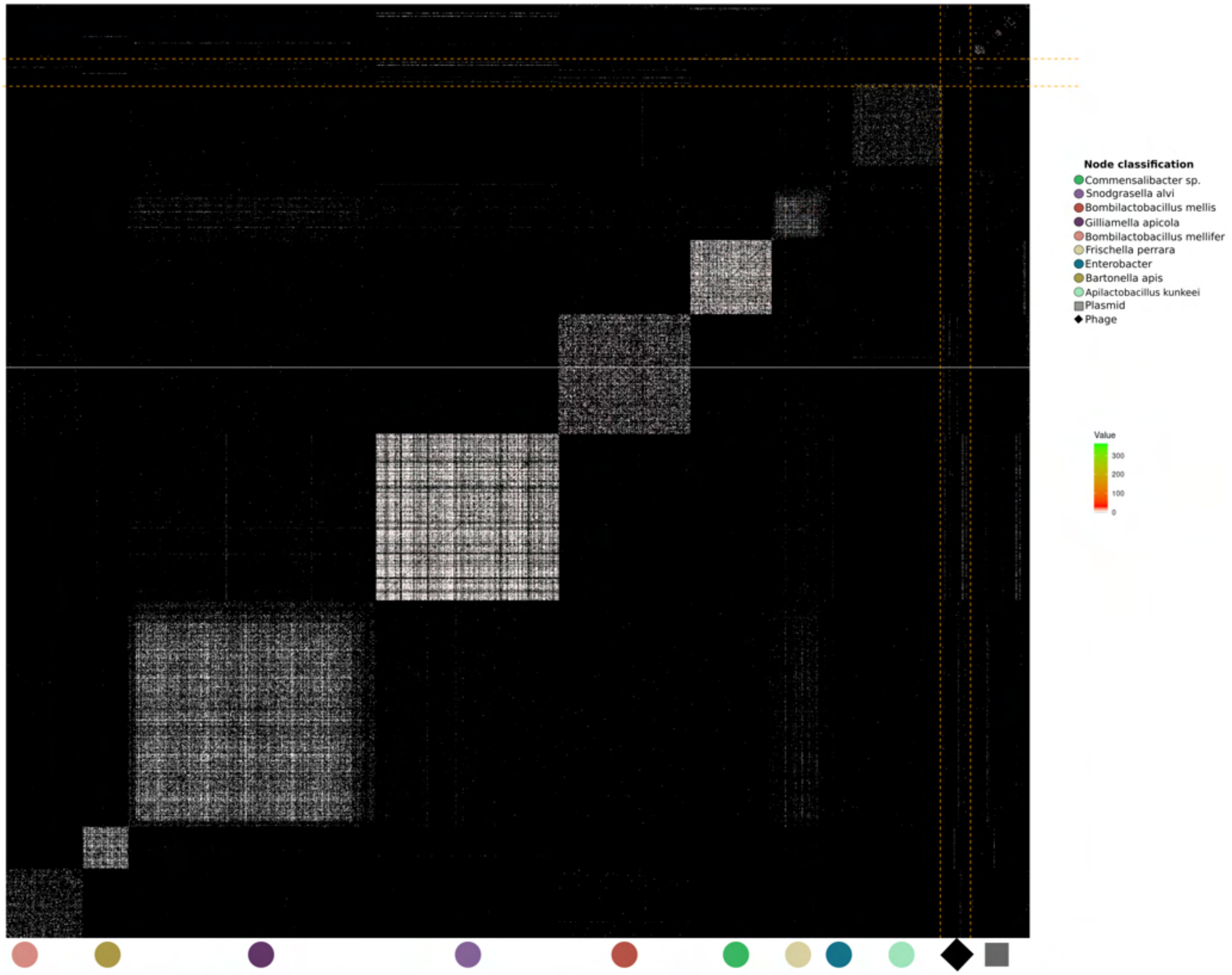


Supplemental Figure 5: Normalized HiC heatmap for Metagenome B. Contig-to-contig interactions with coverage $\geq 1$ are shown in white with more significant interactions appearing red and green. Contigs corresponding mMAG assemblies, vMAG assemblies, and pMAG assemblies are shown on the X-axis.

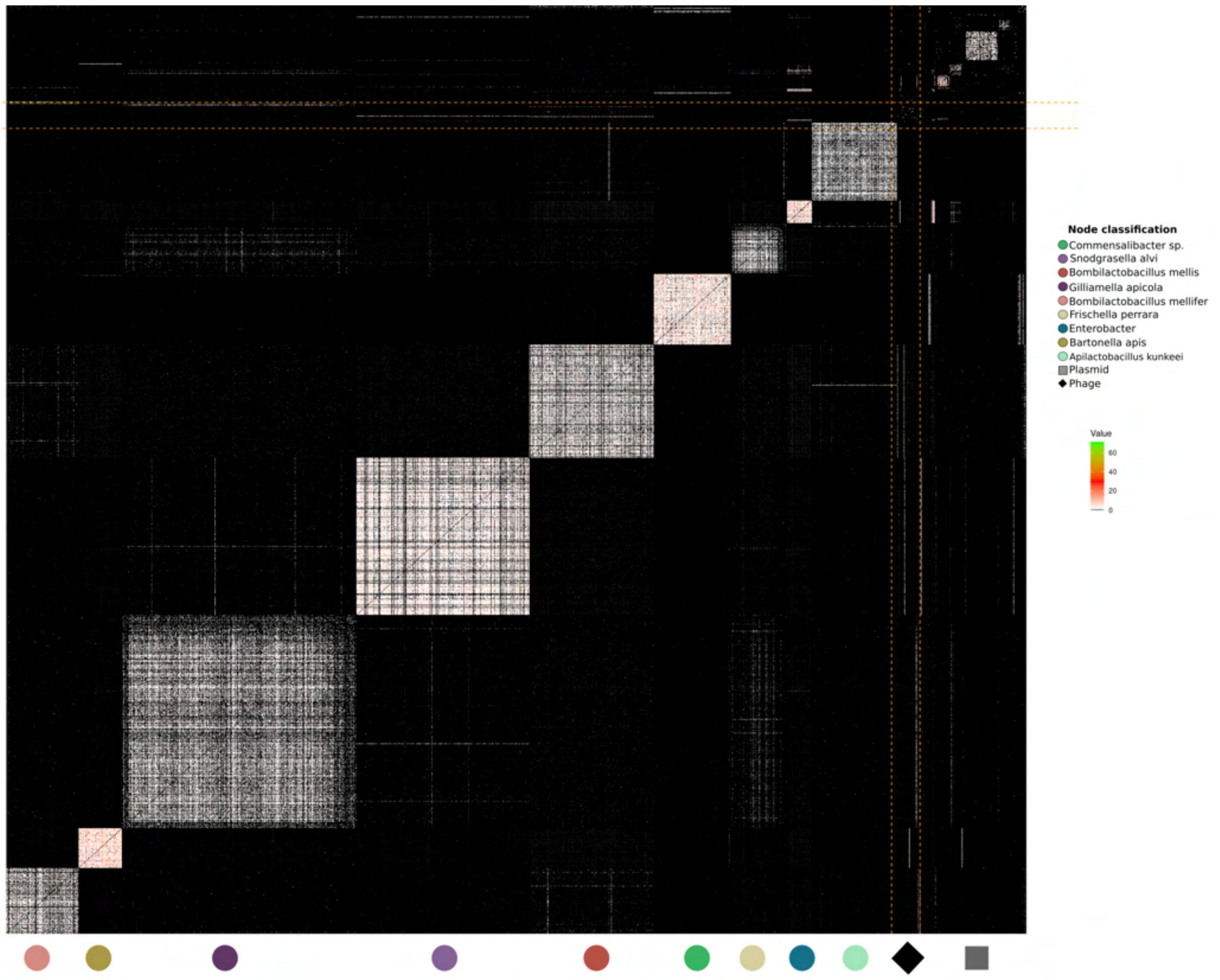


Supplemental Figure 6: Normalized HiC heatmap for Metagenome C. Contig-to-contig interactions with coverage $\geq 1$ are shown in white with more significant interactions appearing red and green. Contigs corresponding mMAG assemblies, vMAG assemblies, and pMAG assemblies are shown on the X-axis.

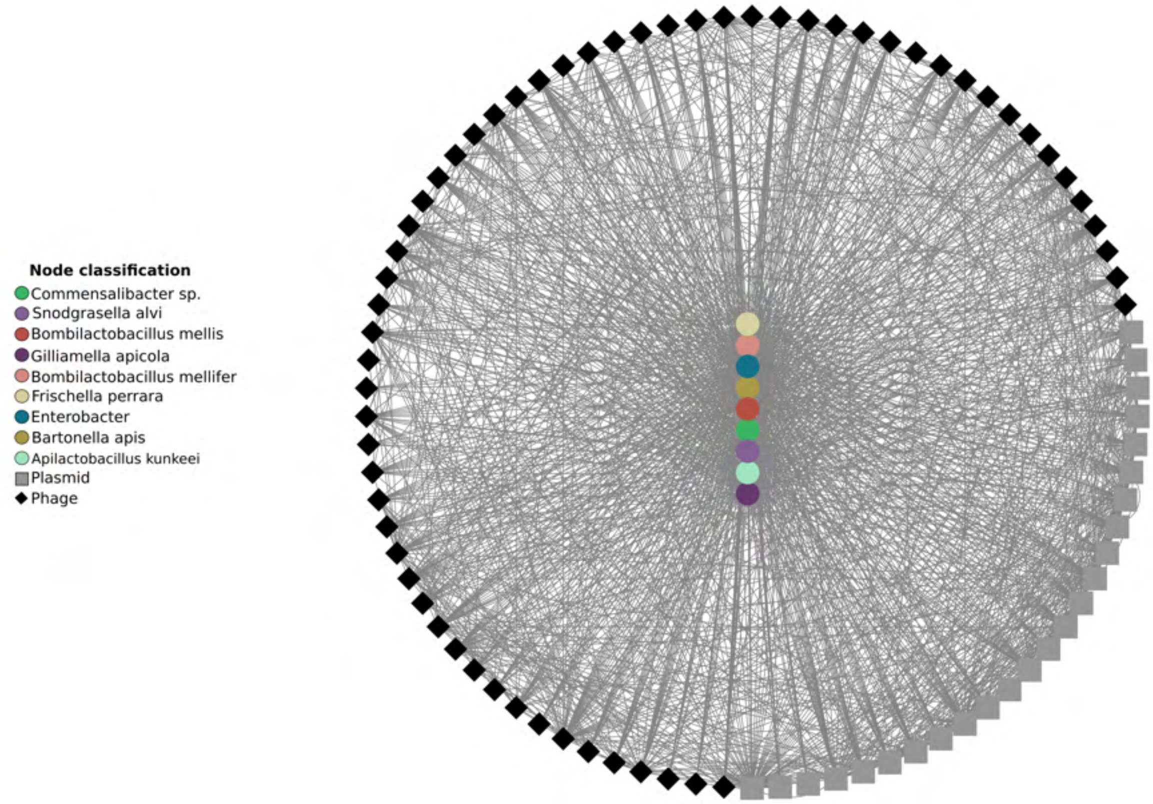


Supplemental Figure 7. Raw HiC network between mMAGs, vMAGs, and pMAGs.

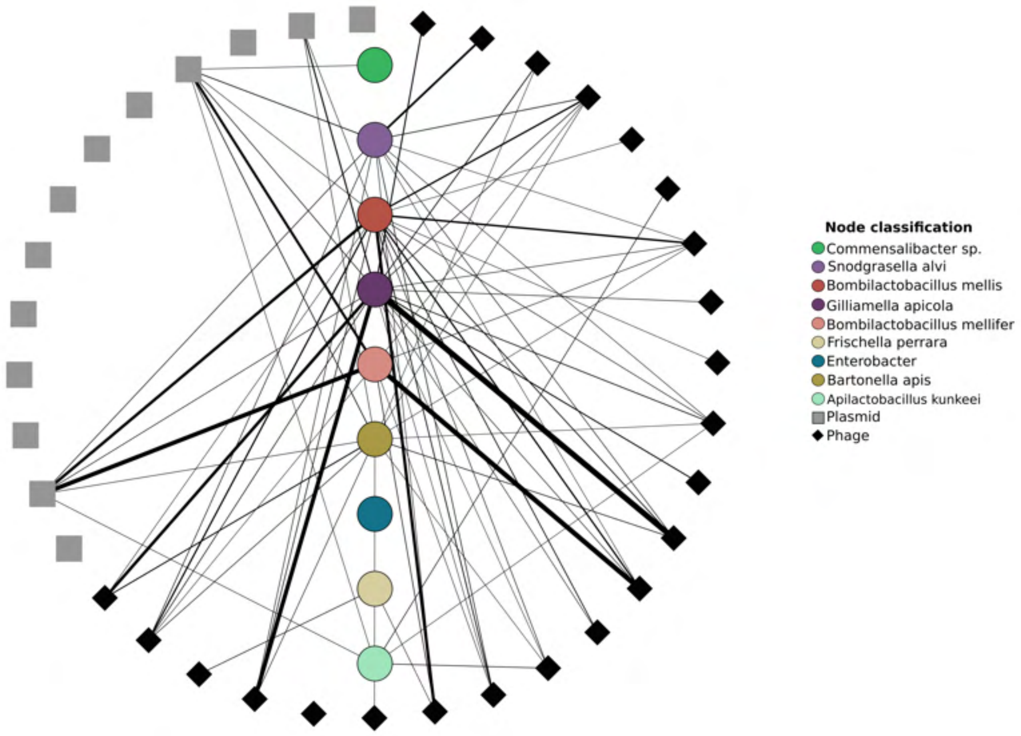


Supplemental Figure 8. Individual normalized HiC network for Metagenome A.

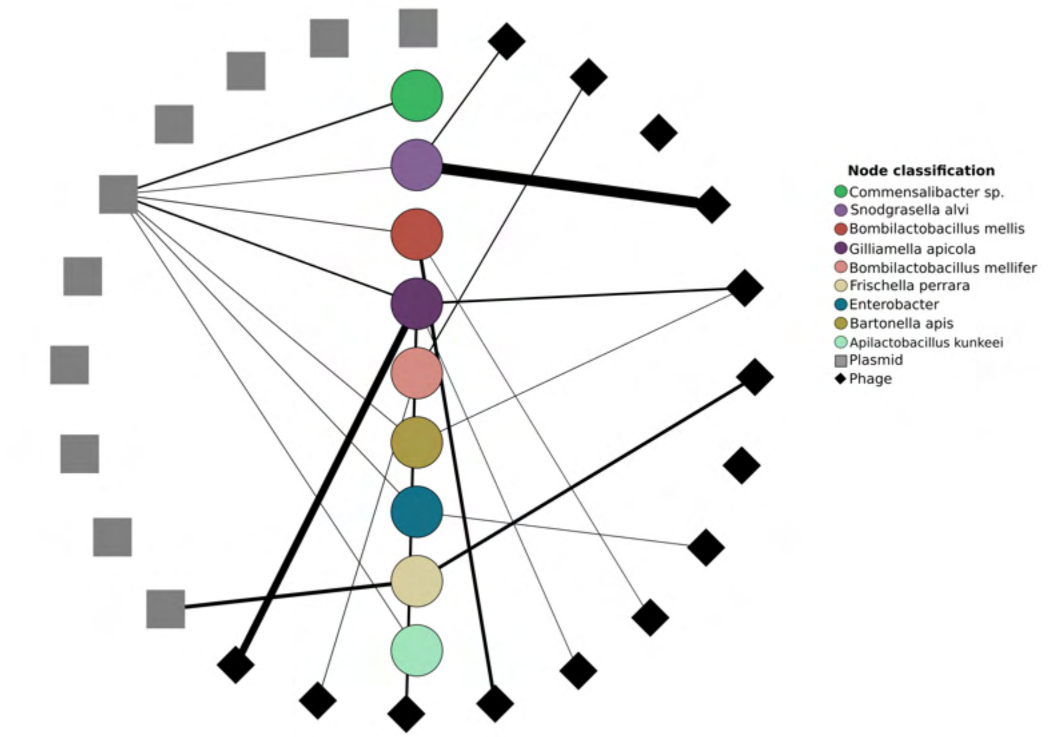


Supplemental Figure 9. Individual normalized HiC network for Metagenome B.

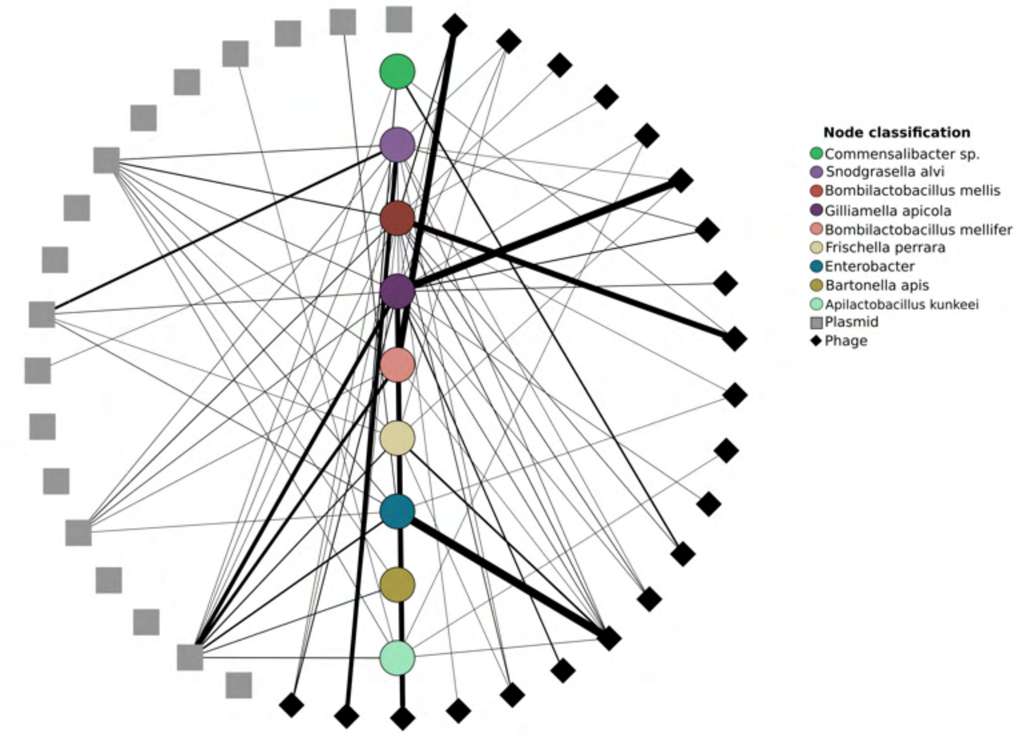


Supplemental Figure 10. Individual normalized HiC network for Metagenome C.

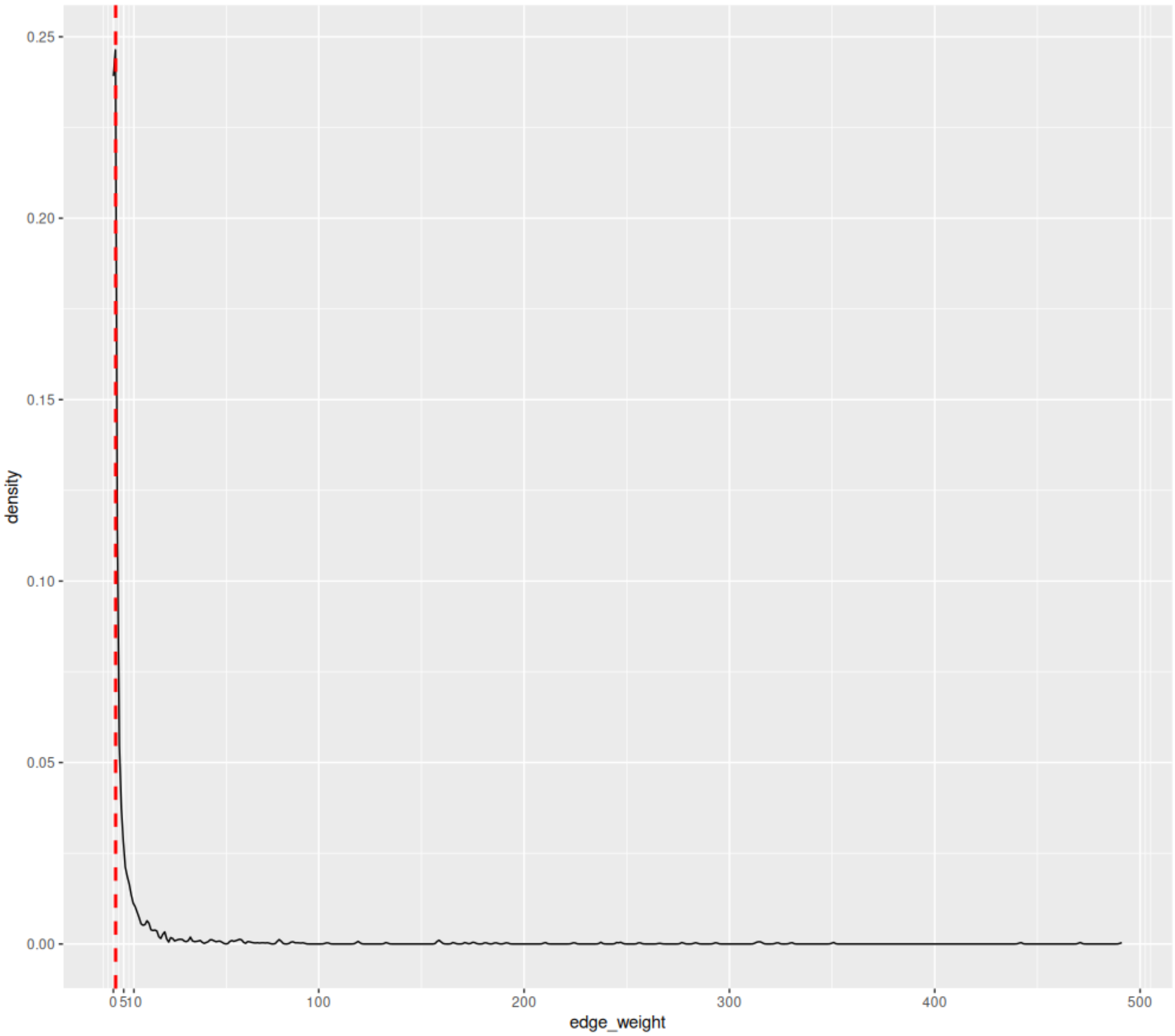


Supplemental Figure 11. Distribution of HiC edge weight (normalized coverage) across all contigs. Red dotted line corresponds to cut-off as imposed by *A. kunkeei*

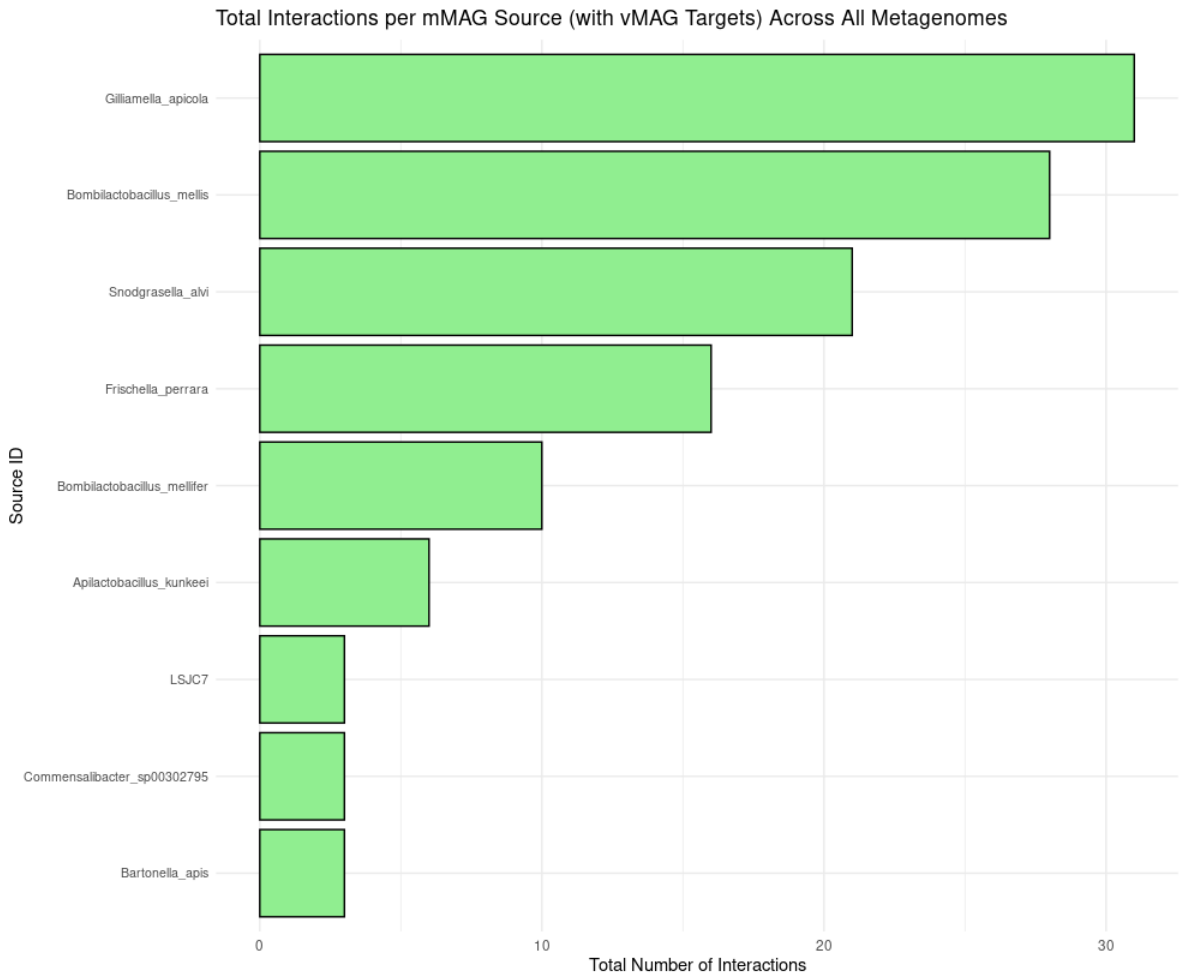


Supplemental Figure 12. Number of unique vMAG x mMAG interactions for each mMAG assembly across all metagenomes. LSJC7 is *Enterobacter*.

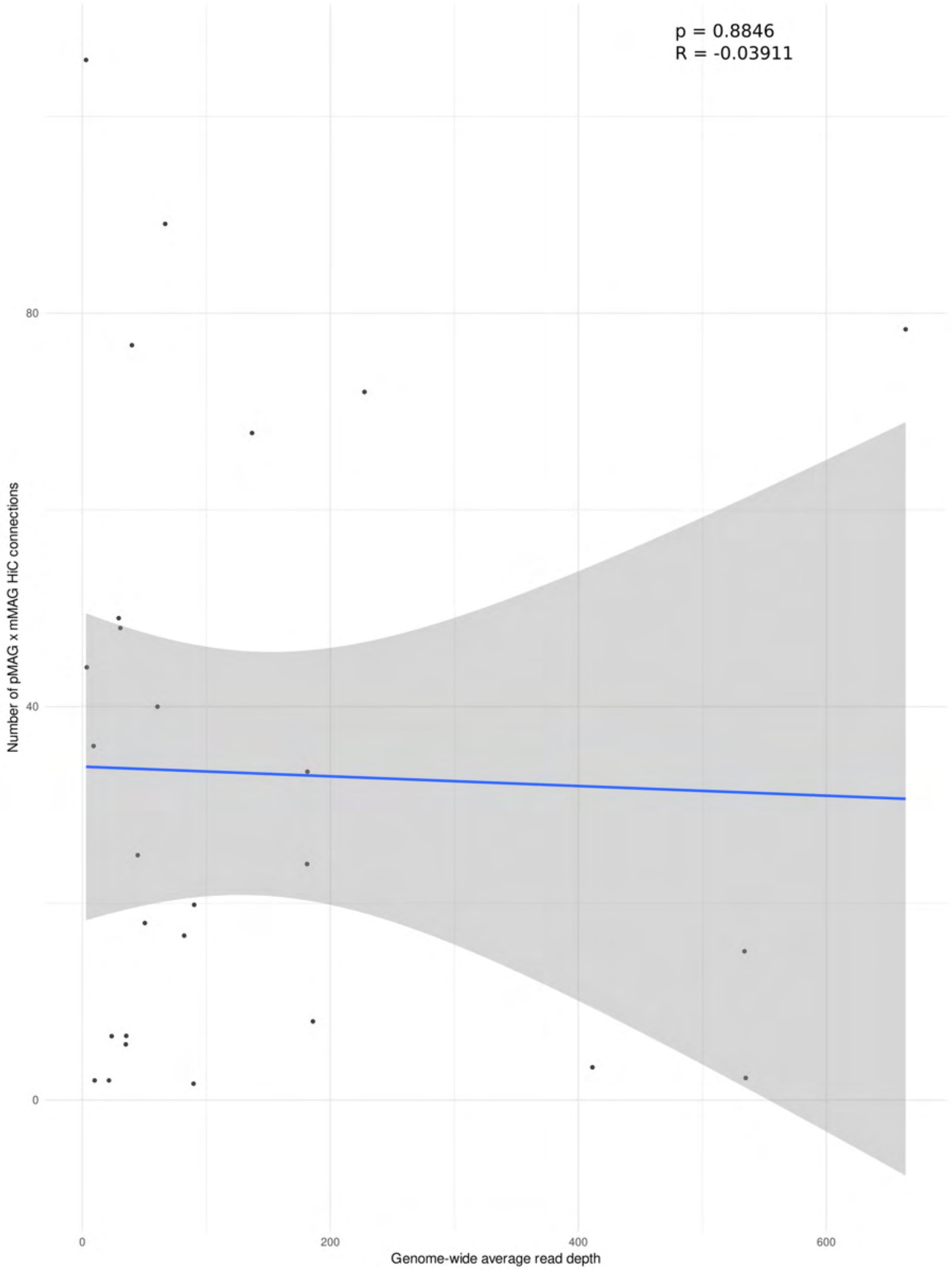


Supplemental Figure 13. Spearman correlation between the number of pMAG x mMAG HiC connections (y-axis) and genome-wide average read depth.

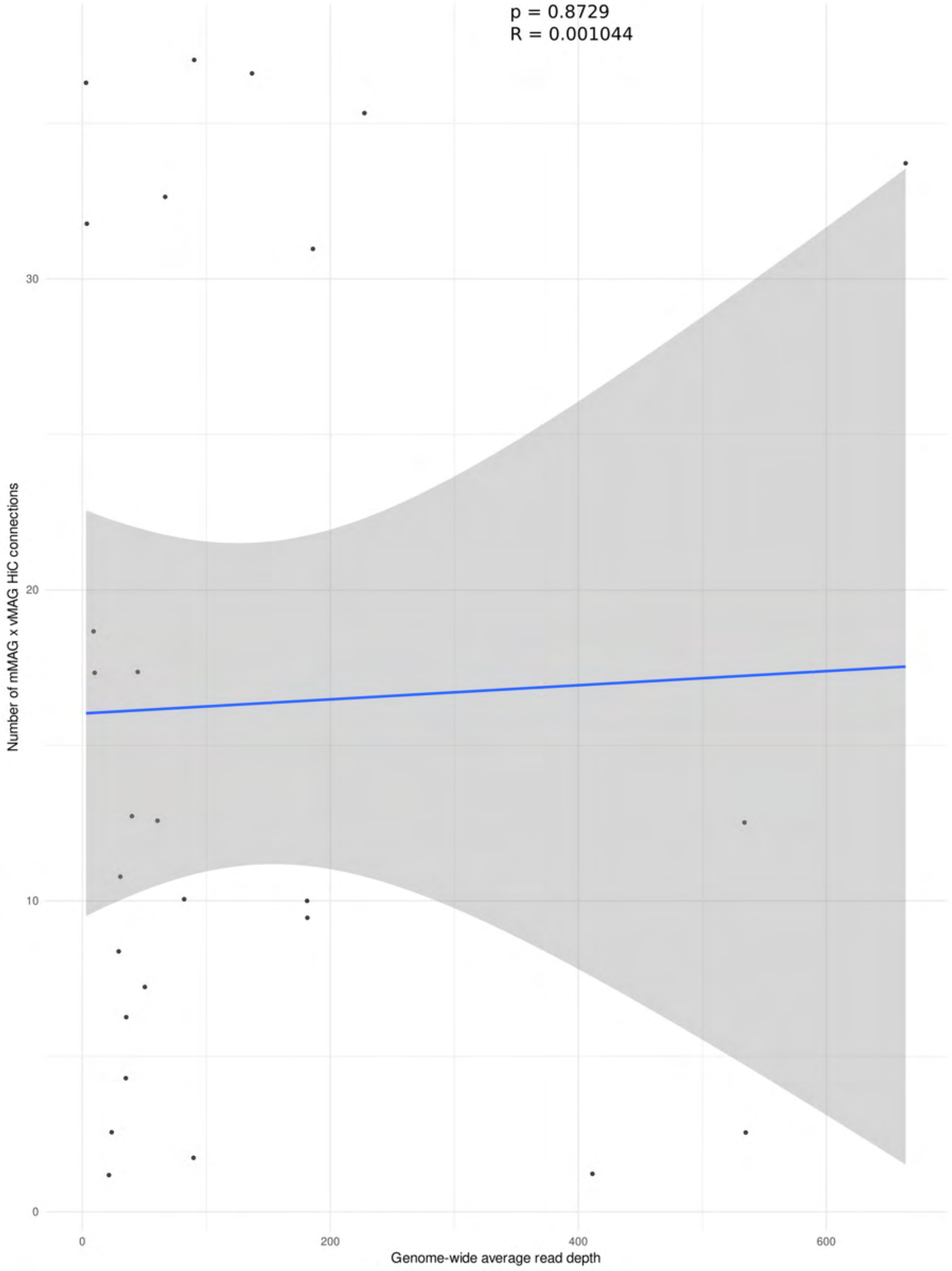


Supplemental Figure 14. Spearman correlation between the number of vMAG x mMAG HiC connections (y-axis) and genome-wide average read depth.
